# Alpha and beta myosin isoforms and human atrial and ventricular contraction

**DOI:** 10.1007/s00018-021-03971-y

**Published:** 2021-10-26

**Authors:** Jonathan Walklate, Cecilia Ferrantini, Chloe A. Johnson, Chiara Tesi, Corrado Poggesi, Michael A. Geeves

**Affiliations:** 1 Division of Natural Sciences, School of Biosciences, University of Kent, Canterbury, CT2 7NJ UK; 2 Department of Experimental and Clinical Medicine, University of Florence, Florence, Italy; 3 Present Address: Cambridge Institute for Medical Research, University of Cambridge, Cambridge, UK

**Keywords:** Heart, Myosin-structure–function, Cardiomyocytes, Cardiac proteins

## Abstract

Human atrial and ventricular contractions have distinct mechanical characteristics including speed of contraction, volume of blood delivered and the range of pressure generated. Notably, the ventricle expresses predominantly β-cardiac myosin while the atrium expresses mostly the α-isoform. In recent years exploration of the properties of pure α- & β-myosin isoforms have been possible in solution, in isolated myocytes and myofibrils. This allows us to consider the extent to which the atrial vs ventricular mechanical characteristics are defined by the myosin isoform expressed, and how the isoform properties are matched to their physiological roles. To do this we Outline the essential feature of atrial and ventricular contraction; Explore the molecular structural and functional characteristics of the two myosin isoforms; Describe the contractile behaviour of myocytes and myofibrils expressing a single myosin isoform; Finally we outline the outstanding problems in defining the differences between the atria and ventricles. This allowed us consider what features of contraction can and cannot be ascribed to the myosin isoforms present in the atria and ventricles.

The human heart is an astounding machine pumping blood round the body to transport oxygen, heat and nutrients to the peripheral tissues and returning with carbon dioxide and other waste products. The heart needs to match the supply of oxygen precisely to the local needs since there is limited storage of oxygen in tissues. To do this, the resting heart beats typically about once per second with each ventricle delivering ~ 70–80 ml of blood at a significant, though quite distinct, systolic pressure into the arteries of the systemic and pulmonary circulation. Between beats, both systemic and pulmonary arterial pressures fall to lower and quite distinct diastolic levels. During systole, the ventricles are delivering blood round the lungs and body and are required to deliver a large volume (70–80 ml/beat ~ 4–5 l/min) of blood at high pressure (for the left ventricle 120 mmHg, which is 1.5 atmospheric pressure or 20 lb/sq in—approaching the same order as car tyre pressure). During diastole, the ventricles relax and are refilled with the assistance of atrial contraction. During their contraction, the atria deliver a small volume of blood (~ 20–25 ml) at a modest pressure of well below 20 mmHg. The contraction of the two types of heart chambers is quite distinct with different mechanical constraints (see Atrial vs. ventricular chamber mechanics below).

Myosin is the protein that is responsible for the generation of the powerful contractions of the heart. In the healthy human heart, the ventricles express predominantly β-myosin while the atria express predominantly the α-isoform. The two myosin isoforms, α and β, although 91% identical in motor domain sequence [1], have distinct mechanical and biochemical properties. Here, we will consider the extent to which the myosin isoforms define the nature of the atrial vs. ventricular contraction and how the properties of the myosin isoforms are optimised for the mechanical task they are required to do. Before doing this, it is salient to outline the differences in the atrial and ventricular contraction cycle.

## Atrial vs. ventricular chamber mechanics

**Fig. 1:**
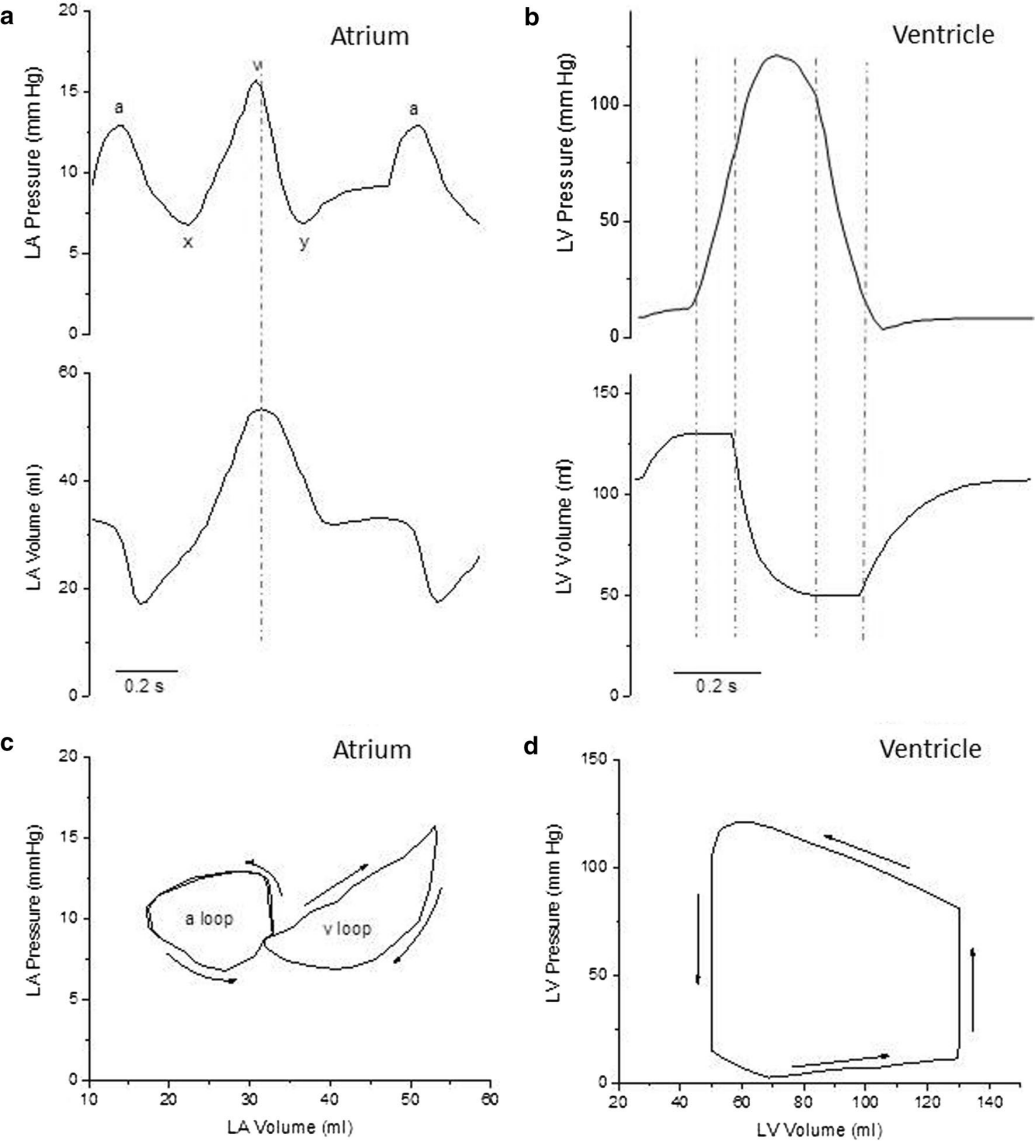
Left Atrial and Left Ventricular Pressure and Volume changes during the cardiac cycle. **a** Time course of the changes of Left Atrial (LA) Pressure (top) and Volume (bottom) during a cardiac cycle. The vertical dashed line indicates the time of mitral valve opening. ‘a’ atrial pressure increase during atrial systole; ‘x’ atrial pressure decrease during the rapid ejection phase of LV systole; ‘v’ atrial pressure increase during the slow ejection phase and the isovolumic relaxation of the LV; ‘y’ atrial pressure decrease following mitral valve opening. The figure is based on those in Stefanadis et al., Rosca et al., and Pironet et al. (REF [5], [9], and [15]) **b** Time course of the changes of Left Ventricular (LV) Pressure (top) and Volume (bottom) during a cardiac cycle. Vertical dashed lines indicate (from left to right) the time of mitral valve closure, aortic valve opening, aortic valve closure, and mitral valve opening. **c** and **d** Pressure–Volume loops of the LA (C) and LV (D): Arrows indicate the direction of the P–V loop as a function of time

The active and passive tension and the length changes of atrial and ventricular cardiomyocytes (composing the muscle walls of atria and ventricles) during a cardiac cycle determine the pressure and volume changes of the heart chambers responsible for the pump function of the heart. The typical time evolution of left atrium (LA) and left ventricle (LV) pressures and volumes during a cardiac cycle are shown in Fig. 1a and b. When atrial and ventricular pressure and volume are plotted against one another during a full cardiac cycle, the result is a closed curve in the pressure–volume plane, called the pressure–volume (P–V) loop. An example of such P–V loops for LA and LV is given in Fig. 1c and d.

The ventricles function as intermittent pumps that drive blood flow through the vessels to the pulmonary and systemic circulation. Both pumps develop the pressure necessary to open the semilunar valves under quasi-isometric conditions during the isovolumic phase of systole. A bolus of blood is then ejected (stroke volume) against the increasing pressures of the pulmonary artery and aorta, also providing some kinetic energy to the ejected blood. The duration of ventricular systole is around 300 ms at the normal heart rates of the resting body conditions. The stroke volume of the two ventricles is the same (70–80 ml in humans), while the pressure imparted by the LV to the ejected blood is much higher (80–120 mmHg) than that of the right ventricle (10–25 mmHg). This is due to the large difference in the resistance to blood flow offered by the systemic and pulmonary circulations. The area of the LV P–V loop (Fig. 1d) accounts for both the extent of the stroke volume and the amount of pressure imparted by LV to the stroke volume, and is a measure of the stroke work of the LV that is related to the oxygen consumption of the cardiac chamber. Because the LV stroke work represents most of the overall cardiac work and accounts for a great deal of the overall energy consumption of the heart, most research has been focused on trying to understand the ejection capabilities of the LV during systole. As a result, little attention has been paid to the heart chamber located upstream, namely the LA. More recently, the study of the heart’s diastolic behaviour, the way in which the heart fills, has gained interest since impaired filling implies impaired ejection. This direction naturally led researchers to focus on the LA and on the way it modulates LV filling [2, 3]. Study of the LA mechanical function can be performed using a variety of measuring techniques, such as echography, angiography, computed tomography, magnetic resonance imaging and invasive catheterization [4]. The importance of the LA as a mechanical chamber is now well recognised. It is supposed to empty the blood it receives from the pulmonary veins into the LV within a narrow pressure range of 8–14 mmHg within a fraction of a second. Even a slight increase in LA pressure may result in pulmonary venous regurgitation and the incoming venous tides are reversed, which results in the so-called pulmonary congestion.

During a cardiac cycle, the LA performs three different roles, all aimed at LV filling; they are the reservoir, conduit, and booster pump functions [2, 5].

First, during LV systole and isovolumic relaxation, when the mitral valve is closed, trans-mitral flow is zero and the LA provides a reservoir that receives blood from the pulmonary veins and stores energy in the form of pressure. The reservoir phase of the LA cycle leads to what is referred to as the ‘‘v’’ wave portion of the LA pressure tracing. The reservoir function of LA is modulated by LV contraction, through the descent of the LV base during LV rapid ejection phase (that is responsible for the ‘x’ wave in the LA pressure record), by the right ventricular systolic pressure transmitted to the LA through the pulmonary circulation, and by LA mechanical properties (i.e. chamber compliance).

After the mitral valve opens, the LA behaves as a conduit to fill the LV. Blood is passively transferred into the LV through the LA via a small pressure gradient during the rapid filling phase of diastole and flows passively, in very small amounts, from the pulmonary veins into the LV during diastasis. The conduit function, aided in the rapid filling phase by the increased return of blood from the pulmonary veins due to the “y” wave portion of the LA pressure curve, is modulated especially by the LV diastolic mechanical properties.

At the end of LV diastole, the LA actively contracts as a booster pump to further fill and pressurize the LV in less than 100 ms. The pump phase of the LA cycle leads to what is referred to as the ‘‘a’’ wave portion of the LA pressure curve. Atrial contraction is an auxotonic type of contraction against increasing, but always rather low, loads. LA systole increases the LV end-diastolic volume by 15–30% and the stroke volume of the next ejection by 20–30% in normal subjects and substantially more in the presence of impaired LV relaxation. Atrial booster pump function has been estimated by changes in cardiac output and LV diastolic volume when atrial systole was either absent or improperly timed [6, 7], and by relative LV filling as estimated with several approaches (studies quoted by [8]). LA booster pump function reflects the magnitude and timing of atrial contraction and is modulated by LA intrinsic contractility, LV compliance, LV end-diastolic pressure (atrial after-load), and by the degree of the venous return (atrial pre-load) [9]. Although it is unclear how effective the heterometric regulation of atrial contraction is compared with ventricular contraction (e.g. Korte and MacDonald 2007) [10], it has been demonstrated that the Frank–Starling mechanism is also operative in the LA [11]. LA output increases as atrial diameter increases, which contributes to maintaining a normal stroke volume [9]. Moreover, LA contractile function may decrease in the presence of severe LA dilation [9], though it is unclear whether this may occur because the declining phase of the length–tension relationship is reached. In fact, the peak of the active force–sarcomere length relationship is likely shifted in heart muscle to longer sarcomere lengths than expected from a reduction in the overlap between thin and thick filaments [12]. Thus, the study of LA function can provide additional information, incremental to simple LA volume measurement.

The evaluation of the P–V curve is the most accurate and representative index for characterising LA mechanical function in different haemodynamic conditions [9, 13–15]. The P–V relationship is depicted in Fig. 1c. Arrows indicate the direction of the P–V loop as a function of time. The curve forms a double loop, giving it a particular figure-eight shape. The right lobe of the curve (the one at higher volumes) is the ‘v’ loop that represents the passive properties of the atrium, namely the reservoir and conduit functions and corresponds to LA passive filling and emptying, the latter during the rapid LV filling phase. During diastasis, there is little change in the LA volume while LA pressure increases. The left lobe of the LA P–V curve (the one at lower volumes) is the ‘a’ loop, which is caused by active contraction of the atrium. It may be surprising that in the LA the ‘v’ loop, that is a passive volume-dependent loop, generates more pressure than the ‘a’ loop, which is an active pressure-dependent loop. In the right atrium (RA), that exhibits a qualitatively similar P–V loop during the cardiac cycle, pressures are in a lower range (2–6 mmHg) compared to LA and, at variance with LA, the ‘a’ loop is usually more prominent and generates more pressure than the ‘v’ loop. The ‘v’ wave is more prominent in LA than in the RA. ‘V’ waves are passive atrial filling waves and are timed during ventricular systole. The LA wall is relatively thicker than that of the RA and is a relatively stiffer chamber. Apart from a relatively thinner wall, RA size is more than that of LA; hence, it can accommodate more volume raising its pressure less. The LA is decompressed by relatively stiff pulmonary veins with a mean pressure of 8–10 mmHg that cannot adequately dampen the refluxing tides of ‘v’ waves, while the low-pressure venae cavae of the RA may dampen the RA ‘v’ waves more easily. Finally, the adjoining systemic LV adds up to the stiffness of LA filling.

As a consequence of the biphasic mechanical behaviour of atrial chambers during the cardiac cycle, flow through the mitral valve, measured by Doppler echocardiography, also exhibits a biphasic behaviour [16, 17]. When the mitral valve is completely closed, trans-mitral flow is obviously zero. During the first early, passive phase of ventricular filling, trans-mitral flow exhibits a peak, termed the ‘‘E wave’’. Then, during diastasis, when the LV is relaxed and before the LA contracts, the trans-mitral flow is nearly zero. Finally, when the LA actively contracts, trans-mitral flow peaks again. This second peak is called the ‘‘A wave’’.

In summary, LA twitch contraction must be very fast (100 ms in humans, a few tens of ms in rodents) to complete LV filling before the start of the much longer LV systole. LA systole occurs under mechanical conditions that are quite different from those of LV systole; LA cardiomyocytes rapidly shorten against the relatively low LV end-diastolic pressures. A very fast atrial twitch that empties the blood into the ventricles becomes even more important to ensure adequate filling at high heart rates when the time for passive ventricular filling is reduced. LV twitch contraction needs much more contractile strength and lasts much longer to ensure that about 60% of the LV end-diastolic volume is ejected against the rather high aortic pressure. Thus, to optimise human cardiac contraction in the LA compared to LV different contraction speeds, different power outputs and potentially different energy economies are required. As we will show in the following sections, expression of different myosin isoforms can provide some but not all of the features required. Additional specific properties of atrial tissue, mostly independent from the features of the molecular motor expressed in the cardiomyocytes, also affect the pump, reservoir, and conduit functions of the atria and are relevant to determine an efficient mechanical performance of the heart. Interest in the relationship between myosin motor kinetics and twitch dynamics is not new. In 1967, Barany [18] published a comparative study of different muscles from a broad range of species and showed that isometric twitch duration was correlated closely with myosin ATPase activity. These landmark findings were subsequently substantially confirmed [19, 20].

## Myosin isoforms in the heart

**Fig. 2:**
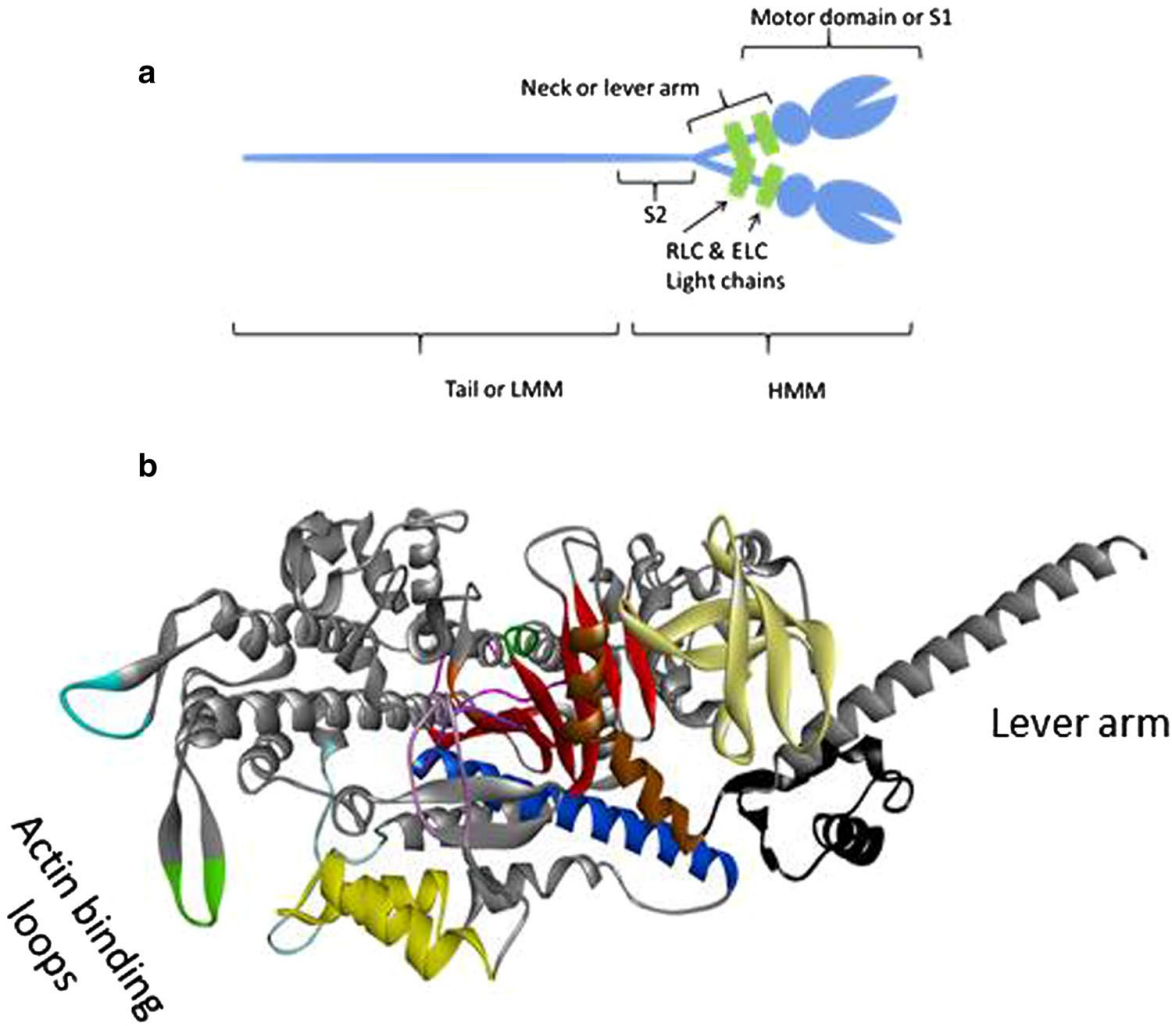
Structure of myosin. **a** The blue represents the two myosin heavy chains which make up the bulk of the myosin molecule. The long tail or Light Meromyosin (LMM) is the C-terminal part which forms a coiled coil dimer. The molecule can be split into two proteolytically, to form LMM and Heavy Meromyosin (HMM), HMM can be further split into sub-fragment 1 (S1)—the globular motor domain and S2 a short-coiled coil dimer. Depending upon the site of cleavage, S1 can include one or both of the small light chains (Essential Light Chain and Regulatory Light Chain) which bind to and stabilise the neck/lever arm. **b** Structure of human β-cardiac myosin homology model with key structural elements highlighted based on the bovine β-myosin in the post-rigor state (PDB: 6FSA). The central seven stranded β-sheet is shown in red. Other structural elements are colour-coded to match Fig. 3

There are two isoforms of myosin found in the human heart commonly referred to α- and β-cardiac myosin. Each myosin is a hexamer made up of two heavy chains (HC) and four calmodulin-like light chains (LC; see Fig. 2). The α- and β-HCs are expressed from different genes, *MHY6* and *MHY7*, respectively, and therefore the protein heavy chains are also known formally as MyHC-6 and MyHC-7. The N-terminal 90 kDa region of the heavy chain folds into a globular motor domain and is followed by a pair of helical IQ domains each of which associates with two LCs and together the IQ domains and LCs are called the neck or lever arm of the myosin (see Fig. 2). The IQ domains are followed by a short α-helical domain, S2, and a longer α-helix domain referred to as the tail. The S2 and the long α-helix regions self-associate to form a coiled coil dimer such that one molecule of myosin consists of two HCs and four LCs. The dimer of two S2 domains together with their motor domains is known as heavy meromyosin (HMM) while the long-tail dimer is light meromyosin (LMM). The LMMs of myosin further self-associate to form the bipolar thick filament. In early literature, the whole myosin isoforms identified in the cardiac ventricle of small mammals were referred to as the V1 to V3 isoforms where V1 and V3 were α- and β-myosin, respectively, and V2 was a mixture of isoforms [21], discussed in more detail below.

**Fig. 3:**
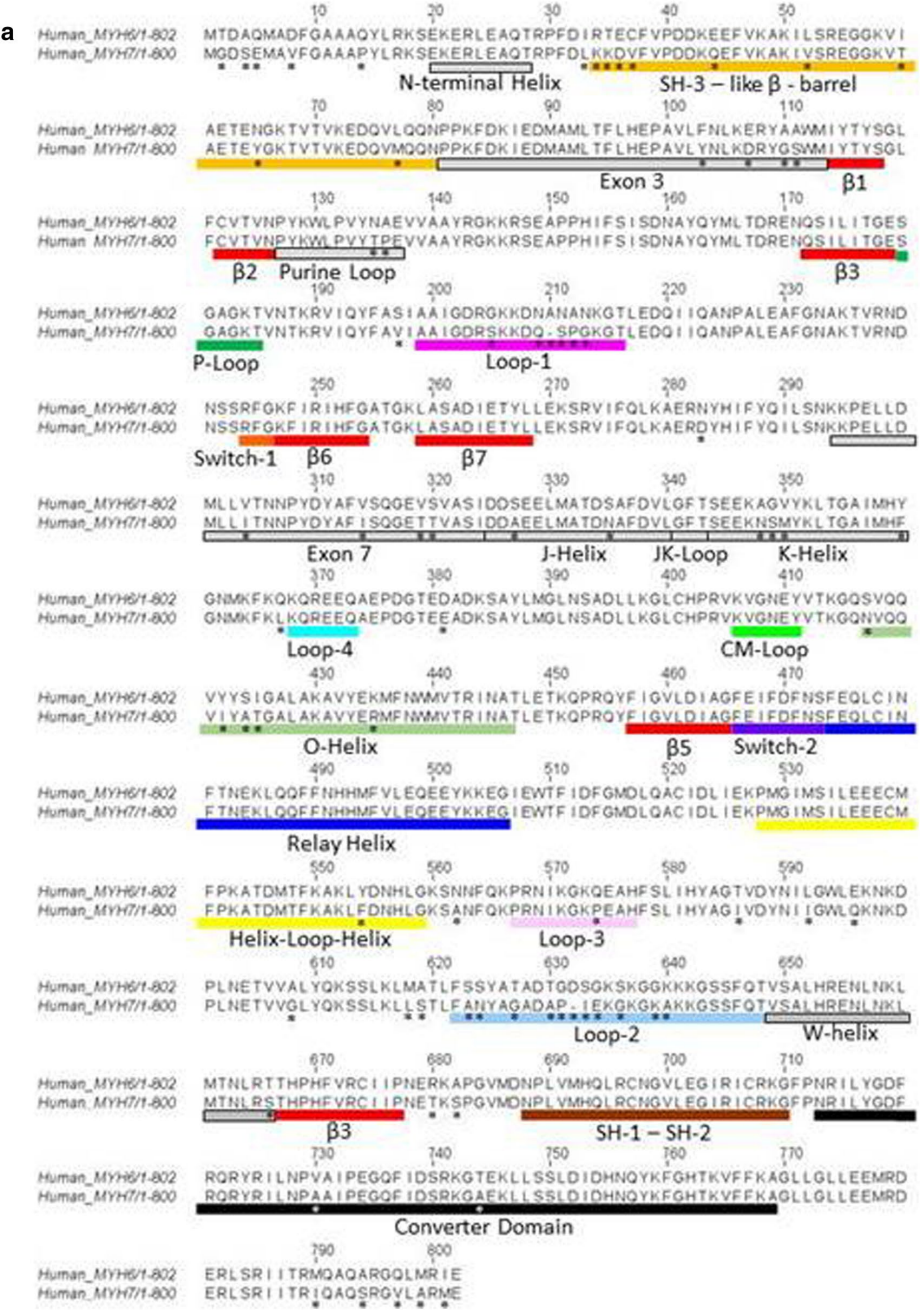

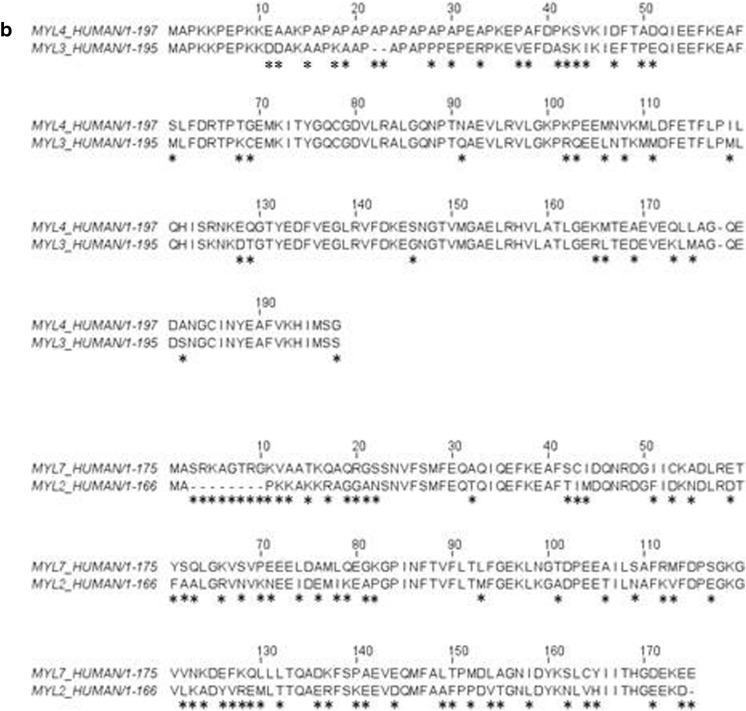
Alignment of human α- and β-cardiac myosin motor domain and LC sequences. **a** The HC alignment with key areas of the structure labelled (UNIPROT code P12883 for MYH7 and P13533 for MYH6). Lines under parts of the sequence have the same colour code as in (Fig. 2). **b** Sequence alignment of LC1a (MYL4—UNIPROT code P12829) and LC1v (MYL3—UNIPROT code P08590) above, and the LC2a (MYL7—UNIPROT code Q01449) and LC2v (MYL2—UNIPROT code P10916) below. The LC1s have a sequence identity of 80.51% and the LC2s sequence identity of 62.05%. * indicates sequences changes between the two sequences

The two LCs that associate with each myosin isoform also differ. As for all muscle myosins, the LCs are called the essential light chain (ELC or LC1) which binds nearest to the motor domain and the regulatory light chain (RLC or LC2), for review, see [20]. The RLC can be phosphorylated to modulate myosin motor activity [22]. The RLC, along with S2 and LMM, can be removed by enzymic digestion of myosin to leave a functional motor domain-ELC complex (known as sub-fragment 1 or S1 [23]) that can hydrolyse ATP and move actin in a motility assay. The ELC can be removed but leaves a very fragile myosin motor domain [24]. The α- and β-myosins have distinct ELC and RLC isoforms known as LC1a/v and LC2a/v, where a and v stand for atrial and ventricular, respectively. It should be noted that the β-myosin and its LCs (LC1v-LC2v) have the identical sequences and are expressed from the same genes in slow skeletal muscle fibres which are also known as Type 1, slow skeletal muscle fibres. Each of the four light chains is expressed from a different gene (LC1v, LC2v, LC1a and LC2a from genes MYL3, MYL2, MYL4 and MYL7, respectively). The two LC1s are 80.5% identical in sequence while the LC2s are more variable with 62.1% identity. Both are considerably more variable than the HCs, which are 93% identical. A sequence alignment of the protein chains is shown in Fig. 3. Although the atrial and ventricular LCs preferentially associate with α- and β-myosin HC, some swapping of LCs may occur (as discussed further below and in Differences between atrium and ventricle contraction in addition to myosin isoforms).

In the human heart, α-myosin predominates in the atria and β-myosin predominates in the ventricles, but the precise amounts of each isoform and their distribution throughout the walls of the two chambers are not well defined. The exact amounts of the minor component can vary depending upon many factors including age, gender and health status. These factors are much better defined in animal models where many more controlled experimental studies have been reported. However, comparisons between model heart systems and the human heart are complicated by the observation that the ratio of α- and β-myosin is distinct for each mammal with species size, which is linked to heart rate, being a major factor [25]. Smaller mammals have an increasing amount of the α-isoform in the ventricles of adult hearts. For example, the amount of α in the ventricle/atria is 100%/100% in mice [26], 90%/99% in rats [27–29], and 5%/75% in humans [30–32]. In addition to the variation in the ratio of the two isoforms, each isoform shows significant variations between species. These issues are discussed in relation to motility assays and myofibril studies in Isolation of α- and β-myosin and in vitro studies of the proteins and Structural differences between α- and β-myosin, but in general, all kinetics (ATPase activity, shortening velocity or in vitro motility velocity and a range of mechanical parameters) are faster in small mammals than in large mammals (see Myosin isoforms in the heart on the structural differences between orthologous myosins).

The complexity of isoform expression is illustrated by studies of the rat heart where the proportion of β-myosin decreases from 50% at birth to < 10% by 3 weeks of age [27, 33–35]. Pressure overload also increased the β isoform content [36–38] while exercise reduced β [35], and hemodynamic stress can induce alterations in the pattern of isoform expression [38]. In hyperthyroid rats, the β isoform decreases to zero while in hypothyroid rats, β increases to 100% [27, 28, 39–41]. Furthermore, shifts in isoform expression may also be localised to distinct regions in cardiac muscle. Pagani and Julian showed that the relative amounts of myosin isozymes in the ventricular free walls and papillary muscles may not be identical within the same heart [34]. Subsequent studies from multiple labs demonstrated that the expression of the α isoform in mammalian hearts is more abundant in the epicardium than in the endocardium of the left ventricular mid-wall, demonstrating a gradient in myosin expression [29, 32, 39, 42, 43]. This raises the possibility that the changes in the expression of myosins isoforms reported above could be localized to specific regions of the heart rather than being uniform across the heart. It is assumed that the mixture of α and β isoforms occurs in individual myocytes raising the possibility that αβ-myosin heterodimers can exist in some cells (discussed below). Most of the model systems studied to date focus on either the whole heart or the left ventricle. Much less is known about isoform changes in the atria.

The above section refers to α- and β-myosin distribution, but in most cases, the literature only specifies the HC. Less is known about the LC distribution in the atrium and ventricle. The isoform distribution of both LC1 and LC2, in healthy humans, closely follows the pattern and variability observed for the HCs, with LC1a and LC2a being expressed early in development when α-HC is expressed in both atria and ventricles. Later in development as β-expression increases, then the ventricle LCs expression also increases [44, 45]. Pathological conditions associated with congenital heart disease or ischemia/reperfusion are associated with re-expression of α-HC and atrial forms of the LCs in the ventricle and down-regulation of ventricular LCs [45–48]. As for the atria, very little is known, but upregulation of LC1v and LC2v and down-regulation of LC1a and LC2a, [49] have been reported in association with upregulation of β*-*HC in atrial fibrillation [50, 51]. Overall, the expression of the HC and related LCs appear then to be closely coordinated. Another distinction between the LCs is in N-terminal modification as LC2a is acetylated, while LC1a and both ventricular LC are methylated [52].

## Isolation of α- and β-myosin and in vitro studies of the proteins

Studies of the human isoforms of sarcomeric muscle myosin have been limited by availability of tissue samples. This means that much of our understanding of the two isoforms has come from the study of protein isolated from model organisms, such as mouse, rat, rabbit, pig or bovine heart samples. In addition, until recently, sarcomeric myosins like the cardiac myosins could not be expressed in vitro. This is believed to be because the popular cells used to express proteins (bacteria, insect cells, HEK) lacked key chaperones essential to the correct folding of sarcomeric myosins. The chaperones involved remain to be fully defined [53, 54]. To get round this limitation, Winkleman pioneered the use of a mouse muscle cell line to express the motor domain of human myosin [53, 55]. This has since been used by several groups, most notably by the Spudich and Leinwand laboratories who have expressed the motor domain of each of the 12 human muscle myosin isoforms, including α and β [56, 57]. The expressed isoforms were used to complete single molecule mechanics and detailed biochemical analysis of the major human myosin isoforms together with studies of many disease-associated mutations [58–61]. The approach has now been adopted by a few other groups, but while this remains the best route to express sarcomeric myosin motor domains, the quantities of isolated protein remain modest (a few mg) and it is a slow and expensive process compared to bacterial or insect cell expression systems. More recently, patient-derived stem cells have been used to grow muscle tissue in vitro [62, 63] and myosin can be isolated from such samples but again in limited amounts.

The availability of pure samples of expressed human α- and β-myosin motor domain with just the ELC or both LCs and now HMM has allowed detailed studies of the biochemical and mechanical properties of the two human isoforms [64, 65]. Many myosin isoforms carrying mutations associated with inherited cardiac diseases have also been studied but will not be discussed further here (see reviews [66–68]).

### In vitro studies of human α- and β-myosin motor domains

#### The mechanochemical cycle

**Fig. 4:**
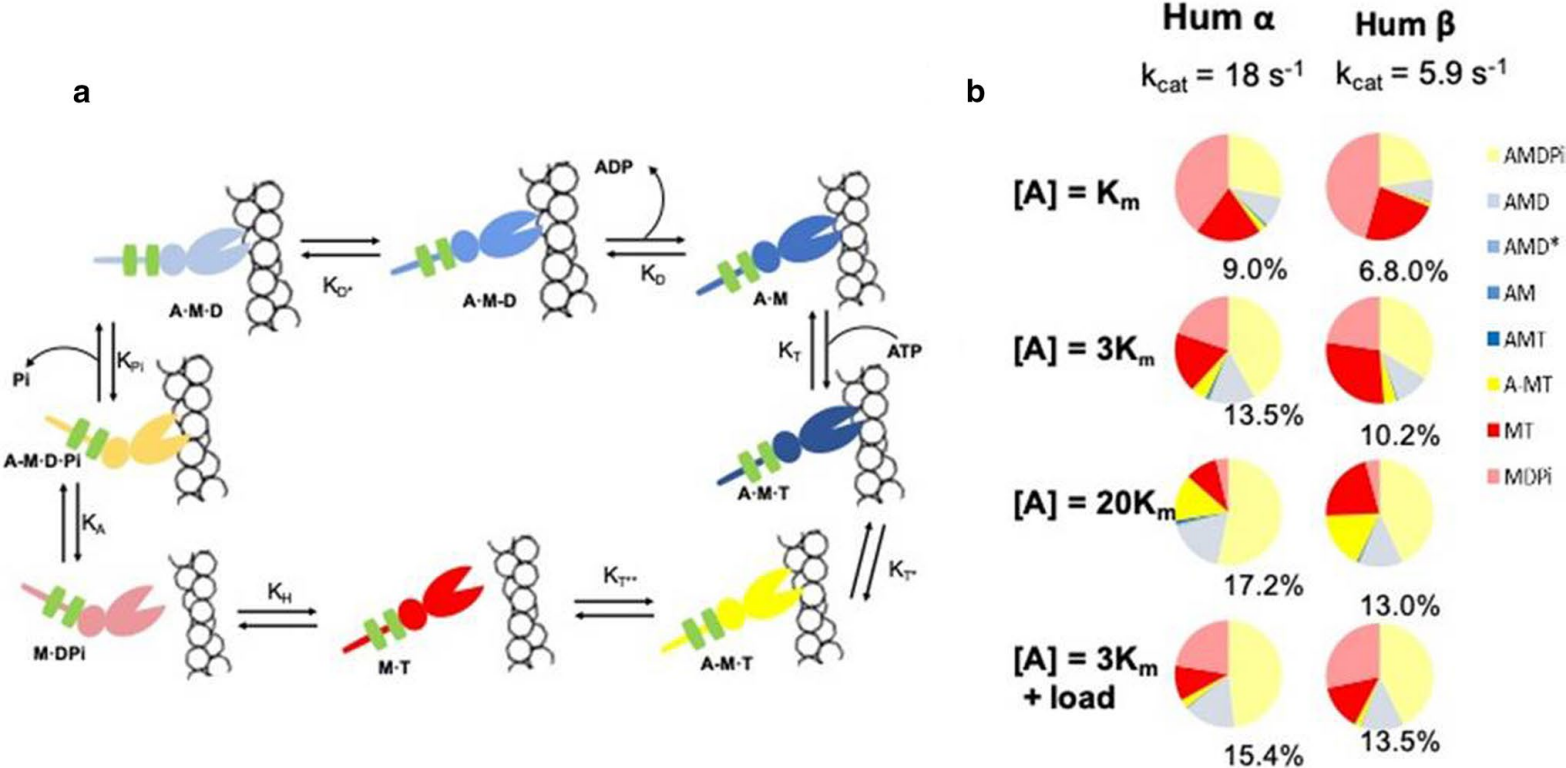
The ATPase cycle of actin–myosin. **a** Actin–myosin mechanochemical cycle. A is actin M is myosin and T, D, Pi represent ATP, ADP and phosphate, respectively. Each step, *i*, of the cycle is labelled with its equilibrium constant, *K*_*i*_ (= *k*_*-i*_/*k*_+*i*_, the ratio of reverse, *k*_*-i*_, and forward, *k*_+*i*_, rate constants). Actin monomers are shown as open circles. The myosin is shown as two ellipses and a linear tail, with the light chains as green rectangles. The colour of myosin indicates the nature of the interaction with actin, red shades are detached from actin, yellow are weakly attached to actin, and blue shades are strongly attached force holding states. Starting from the top left blue A·M rigor state, ATP binds (eq const *K*_*T*_) induces cleft opening to a weakly attached state (yellow, *K*_T*_). Myosin then detached from actin to form M·T (*K*_T**_). The recovery stroke follows accompanied by ATP hydrolysis (*K*_*H*_) which then allows weak rebinding to actin (*K*_*A*_). The power stroke is associated with Pi release and strong binding to actin (*K*_*Pi*_) and precedes two step ADP release, the first associated with an additional movement of the myosin tail or lever arm (*K*_D*_), and then rapid ADP escape (*K*_*D*_) back to the rigor state. b) Fractional occupancies of each state in the ATPase cycle at 3 different actin concentrations, [A] = *K*_m_, 3* Km*, and 20* Km* (where *Km* = [A] required for half of the maximum turnover rate k_cat_) for each isoform and at [A] = 3 *Km* plus 5 pN load. Colours of the pie chart bands match those of (a). Note that the three dark blue states are all very short lived and represent < 1% of the cycle time. The % value next to each chart gives the % of each cycle spent as the force holding A·M·D state. The Figure is adapted from Johnson et al. [75]

All myosins, both muscle and non-muscle, are closely related and all that have been studied to date have a similar actin–myosin cross-bridge cycle, illustrated in Fig. 4a. The basic ATPase cycle of isolated myosin motor domain is slow, typically 0.05 molecules of ATP.s^−1^ per myosin motor domain (i.e. *k*_*cat*_; for review, see [69]; note that all rates and rate constants quoted for the in vitro studies are at 20 °C unless otherwise stated). This is equivalent to the ATPase of a single myosin head in a relaxed muscle where interaction with actin is prevented. The limiting step is Pi release from a stable M.ADP.Pi complex. The binding of this myosin complex to actin accelerates Pi release more than 100-fold and is concomitant with what is called the power stroke —a re-orientation of the converter-lever arm through ~ 120° (see Fig. 4a). This can either generate a force of ~ 5 pN, if contracting against a load (the power stroke), or in the absence of load generate a rapid movement of 5–10 nm at the tip of the lever arm (the working stroke). If the working stroke is completed, then ADP is rapidly released. If the working stroke is prevented by the load, then ADP release is inhibited resulting in a lower ATPase rate under load [70–72]. Once ADP is released, ATP binds rapidly (cytosolic ATP is normally at a concentration of several mM [73]) leading to the opening of the large cleft between the actin-binding sites of myosin and actin dissociation. Once dissociated, the converter-lever arm goes through the recovery stroke accompanied by hydrolysis of ATP. This re-primes the myosin motor ready for another round of actin interaction and force/movement generation.

The above cycle is common for all myosin studied to date, what differs for each isoform is the speed at which each cycle is completed (the ATPase rate) and the fraction of each cycle time the myosin spends in the different states around the cycle. The duty ratio (DR) is defined as the fraction of the total myosin in actin-attached states in the steady-state cycle (= τ/T where τ, is the lifetime of the actin-attached states and T is the total time of each cycle of ATP hydrolysis). All muscle myosins are low duty ratio motors spending at most ~ 10% of the cycle time attached to actin. At one level, this appears to be an inefficient system for force generation, since steady-state force is proportional to the time-averaged number of strongly attached myosin cross-bridges. But it does mean that there is a constant pool of M.ADP.Pi-primed motors available to the muscle, i.e. at maximum activity once a myosin has detached from actin, it may not be needed again on average for another 10 ATPase cycles (10 × τ = T). In fact, when shortening rapidly against zero load, the DR may be much less than 10% and each myosin may contribute once (or less than once) per each heart contraction/relaxation cycle.

At the single molecule level, the step size of a motor can be measured and for all muscle myosins that have been examined, the step size is similar, within the precision of the measurement, at ~ 7–10 nm. The lever arms are all the same size (2 IQ domains and two LC) and the angle through which the lever arm swings is therefore expected to be the same, to give the same step size. Similarly, the force that can be generated in each step is similar, within the precision of the measurement, at 5 pN. If the step size is the same, then the stiffness of the head and the site of any elastic element are likely to be the same.

Note however, although the step size and elementary force generation are the same, the life time of attachment events does vary from isoform to isoform. If DR is constant, then the average number of myosin heads strongly attached remains the same, but for slower myosins, the cross-bridge remains attached for a longer time. Thus, with slow myosins, the ATPase is slower and the energetic cost of maintaining force is lower.

#### Differences between the cross-bridge cycle for α- and β-motor domains

Typically, α and β motor domains differ about two–three-fold in the maximum actin-activated ATPase activity (defined as *k*_*cat*_ [74]), and this is also true of human isoforms [75]. So the ATPase cycle is the same as in Fig. 4a, but an α-myosin completes the cycle 2–3 times faster. If this was the only difference, then the two motors would have similar mechanical characteristics, but the two differ in how much of the total cycle time they spend in each state in the cycle and this results in distinct changes in the mechanics and the efficiency of how the energy of ATP hydrolysis is used [75].

The ability to express pure forms of the cardiac myosin motor domain allowed Deacon et al. [57] to define the rate and equilibrium constants for each of the steps in the cross-bridge cycle as shown in Fig. 4a. While the k_cat_ for the ATPase activity is three times faster for α compared to β isoform, this is not reflected in the individual rate constants round the cycle. Of the 16 rate constants in the cycle (eight forward and eight reverse rate constants), 11 are at least two-fold faster for α-myosin vs β while five remain essentially unchanged. Surprisingly two rate constants are faster for β; the two rate constants for ADP rebinding to AM, k_-D_ and k_-D*_ (see Fig. 4a)_._

The values for each of the rate constants together with the actin dependence of the ATPase rate allowed Mijailovich et al. (2017) to model the complete cycle as a function of actin concentration and predict the occupancy of each of the states during steady-state ATP turnover [75, 76]. The results of the modelling (Fig. 4b) show how the cycle varies for the two human isoforms and can predict differences in the mechanical performance of the two motors. At actin concentrations equal to the value of K_m_ (the actin concentration required for the ATPase rate to be 50% of the maximum, k_cat_/2), the cycle is dominated by the detached and weakly attached states of the cross-bridge (red/pink and yellow shades, respectively, in Fig. 4b), and these are marginally larger for the β (~ 91%) compared to the α isoform (93%), leaving just 9 and 7% as strongly attached states (blue shades), predominantly the A.M.D form. Hence, the duty ratio for the two motors is similar despite the three-fold slower cycling rate for β-myosin. This is as a result of key steps in the attached (Pi release, ADP release) and detached (ATP cleavage) parts of the cycle slowing to maintain a balance between the two parts of the cycle. As actin concentration increases to 3 *Km* (75% of k_cat_) and 20 *Km* (95% of k_cat_), the detached M.D.Pi (pink) state reduces as does the detached M.T (red) state, while the weakly (yellow) and strongly attached (blue) states increase. For the β, the M.T state is larger because the rate of the hydrolysis step is significantly slower for this isoform and now contributes more to limiting the overall k_cat_. Note that the fraction of strongly attached states increases by a factor of 2 for both α and β, maintaining a similar increase in duty ratio as actin concentration increases (0.1–0.19 for α and (0.073–0.14 for β), i.e. both duty ratios increase in parallel. Also, note that the effect of load on the cycle increases the force holding AMD state by 1.9% for α-myosin while it increases by 3.3% for β. The results are plotted at three different actin concentrations (Fig. 4b) because it is not possible to define the effective actin concentrations “seen” by a myosin head in a shortening or isometric sarcomere.

A similar duty ratio is important as this keeps the number of cross-bridges that can hold a load in the steady-state constant. Thus, if the force per cross-bridge is constant (see below), then the similar duty ratio means that myofibrils containing α or β will hold a similar steady-state ensemble force. The small, similar duty ratio also maintains the large pool of detached (M.D.Pi, pink) and weakly attached (A-M.D.Pi, pale yellow) cross-bridges that are available to go through the power stroke and contribute to force generation/force holding as needed.

In addition to predicting a similar steady-state force, the model of the cross-bridge cycle in Fig. 4 allows several other parameters of the mechanical cycle to be predicted. The biochemical cycle in solution is the nearest equivalent to the unloaded shortening muscle fibre where every myosin head has ready access to actin sites and in the absence of any significant load. Under these conditions, the maximum velocity at which myosin will move an actin filament is given by

$$V_0=d/{\tau}=d.{\text{ATPase}}/DR,$$

where V_0_ is the velocity at zero load, *d* is the step size, *τ* is the lifetime of the strongly attached state, DR is the duty ratio, and the ATPase rate is that under the condition where velocity is measured. Assuming a step size of 5 nm, then the modelling predicts a V_0_ of 0.45 µm s^−1^ for α and 0.2 µm s^−1^, for β-myosin. It is also then possible to estimate how much ATP is used per myosin head when moving actin at V_0_. As listed in Table 1, α-myosin uses 0.35 molecules of ATP per myosin head for each nm moved, 2.3-fold more than β-myosin. Thus, α-myosin moves 2.25-fold faster than β but burns up more than twice as much ATP per nm moved, a significant difference in the economy of energy used.

**Table 1:** Predicted mechanical performance for α- and β-myosin based on measured *k*_*cat*_ and differences in the biochemical cross-bridge cycle

Isoform	*k*_*cat*_ (s^−1^)	Vel (µm s^−1^)	% AMD	Fractional ΔAMD with 5 pN load	ATP economy	
					At 5 pN load ATP/s	At max velocity ATP/s/µm
α	18	0.45	13.5	1.15	1.0	0.35
β	5.9	0.2	10.2	1.33	0.35	0.15
α/β	3.05	2.25	1.32	0.86	2.86	2.33

% AMD, fractional change in AMD under load and ATP economy are all predicted at [A] = 3 *K*_*app*_

The effect of load on the rate constant for ADP release has been estimated in single molecule assays for β-myosin but not for α [70, 77]. A 5 pN load slowed down ADP release by ~ a factor of 3 and a similar reduction in the rate of entry into the power stroke (coupled to Pi release) is expected. As there is no effect of load on the detached part of the cycle, the load increases the duty ratio by increasing the population of strongly attached cross-bridges as shown in the last row of Fig. 4b. There are no experimental data for effect of load on the α-isoform cycle. However, if the sensitivity to load of α- is assumed to be similar to that of the β-isoform, then effect of load on the ATPase cycle for the two isoforms can be compared. As shown in Table 1, a 5 pN load increased the fractional occupancy of the force holding AMD state to 1.15 about half as much as that of the β isoform (1.33). The ATP/s usage to bear a 5 pN load was also almost 3 times higher for α compared to the β isoform.

In summary, the modest differences in the amino acid compositions (less than 9%) of the motor domains of the two isoforms lead to quite distinct mechanical cycles. The α-isoform can move in an unloaded system > two-fold faster than the β isoform; thus the cycle of the α-isoform is significantly less affected by load and use > twice as much ATP as the β whether unloaded or holding a force close to that expected in an isometric contraction. In the following sections, we will show that these characteristics are shared by myofibrils and myocytes expressing the different isoforms. This will be followed by exploring how the two isoforms are suitable for the distinct roles in the contraction cycle of the atria and ventricles.

#### Motility assays

In vitro motility assays measure the speed at which myosin will translocate an actin filament over a coated, microscope slide coverslip [78]. The measured velocity of movement or sliding velocity is approximately the equivalent to the unloaded velocity of shortening of a muscle fibre [79]. Numerous studies have demonstrated that sliding velocity varies not only between the 2 cardiac isoforms, but also between different species as is also true of skeletal muscle isoforms [79, 80]. At 30 °C, human α-myosin has a sliding velocity of 2.0 μm/s, compared to β-myosin which ranges from 1.2–1.5 μm/s [81–83]. The measured velocity for β-myosin has a strong temperature dependence, with recent studies showing values ranging between 0.4 and 0.6 μm/s at 25 °C [59, 60, 84]. In common model organisms, the sliding velocities are different compared to human, but the ratio of α:β velocities remains consistent across species at about 2.1–2.4. For example, in rabbit, α-myosin has a velocity of 2.8–4.6 μm/s, in pig 3.8 μm/s and in mice 5.5 μm/s [80, 83, 85, 86]. Rabbit β-myosin has a velocity of 1.1–1.8 μm/s, pig 1.8 μm/s and mouse 2.6 μm/s [26, 80, 83, 86].

## Structural differences between α- and β-myosin

The differences in the ATPase, biochemical cycle and motility described above are due to changes in the sequence of the two isoforms. While there are a few crystal structures of β-myosin motor domain (eg PDB files 6FSA, 4P7H, 4DB1 and 5N69), there is none of the α-isoform. However, because the overall sequence identity of the two isoforms is high at 93.2% (slightly lower in the motor domain, 91%, than the tail, 95%), few changes in the overall fold of the protein are expected. For example, smooth-muscle myosin has the same fold with only 40% sequence identity [87]. The 91% identity in the motor domains translates to ~ 76 changes in sequence in the motor and neck domains. The locations of the sequence changes are illustrated in Fig. 3, which assigns each sequence change to the different regions of the motor domain. This analysis extends an earlier comparison of α and β sequences, completed before any myosin high-resolution crystal structure of myosin was available [88]. The changes are scattered throughout the structure, but there is a slight concentration of sequence changes in the two surface loops (Loop 1 and Loop 2) known to be hypervariable across the wider myosin family [89]. There are six-sequence changes in the 18-residue Loop 1 which is found at the entrance to the nucleotide pocket. This Loop is alternately spliced in some muscle myosins (e.g. tonic and phasic chicken, smooth-muscle myosins [90], scallop, striated and catch muscle myosin [91, 92]), to modulate the ADP affinity to actomyosin. Variations in the sequence of Loop 1 have been introduced in some easily expressible myosins to explore the role of the structure of the Loop (smooth muscle, myosin II, mammalian Myosin 1b, 1c [93–98]), but not in a cardiac myosin. The experimental data suggest that the flexibility of the loop may affect nucleotide binding in the pocket. Both α- and β-isoform loops contain three flexible glycine residues but the position of one glycine is different. A five-residue sequence in Loop 1 is altered from NANAN in α-myosin to the one residue shorter QSPG in β-myosin. The shorter loop in β and the introduction of a proline could make the β loop less flexible and hence contribute to the slower ADP release.

There are 11 changes in the 27-residue Loop 2 which bridges between the upper and lower parts of the 50 kDa domain of the motor. Loop 2 forms part of the actin-binding site (together with Loop 3, Loop 4, the Cardiomyopathy Loop and the Helix–Loop–Helix motif, H–L–H). The structure of Loop 2 is often not well defined by crystallography or cryo-electron microscopy [64, 99–101] suggesting that the loop is flexible and retains some flexibility even when bound to actin in the rigor complex. The net positive charge in the Loop is thought to be an important part of its interaction with the negative charge on the N-terminal region of actin and introducing additional charge into the Loop can increase the rate of binding to actin and the affinity [102]. However, there is little net charge change between the α- and β-Loop 2 (5 lysines and 2 acidic side chains in each) although the position of the charges is slightly different. Exploring variations in Loop 2 in chicken smooth muscle and *Dictyostelium* non-muscle myosin II also indicates that Loop 2 can alter the k_cat_ of the actin-activated ATPase reaction [103, 104]. Risi et al. [100] compared their β-myosin Loop 2 structure with that of fast muscle and non-muscle myosins in which Loop 2 is better resolved. They speculated that loss of the Loop 2 interaction with actin could play a role in producing a slower β-myosin. However, as mentioned above, there are only small changes between Loop 2 in α- and β-myosin and the residues at about half the positions in Loop 2 of both α- and β-myosin vary between mammals, although changes in charge are rare. It is therefore difficult to assign a specific role to residues in Loop 2 without a structure of the α-myosin in complex with actin to compare with β-myosin. In addition, the rigor structure alone does not reveal much about the role of Loop 2 during the process of binding to actin.

In contrast to Loop 2, the other actin-binding loops show only small changes. There is a single change in Loop 3 (Q to P, which is likely to reduce Loop 3 flexibility), one in the H–L–H motif (Y to F), both in the lower 50 kDa domain, and one at the start of Loop 4 (Q to L). The cardiomyopathy loop has no changes. These changes in the actin-binding loops are of interest as the affinity of the motor domain is 2–five-fold tighter for the β-isoform in the rigor complex and ~ two-fold tighter in the steady-state actin-activated ATPase assay [75, 105]. The 2–2.5-fold change in affinity for actin is unlikely to be attributable to the sequence differences in one of the loops but each loop is likely to make a partial contribution.

Loop 3, as seen in the actin.β-myosin cryo-electron microscopy images of Doran et al. and Risi et al. [99, 100], is further away from actin than seen in other skeletal muscle and non-muscle rigor complexes [106–108]. Risi et al. note that there is little meaningful contact with actin at this site which then questions if Loop 3 plays any role in the actin–myosin interface for β-myosin. But the Loop was only partially resolved in the Risi et al. structure allowing for some flexibility of Loop 3. It remains unresolved if Loop 3 could make contact with actin in other stages of the cross-bridge cycle.

There are 7 changes in the first 34 residues at the N-terminus and a further 8 in the following 45-residue SH-3-like domain (residues 35–80). Both regions show significant variation amongst both paralogues and orthologues of muscle myosin IIs. The role of this region in the mechanochemical cycle has been of interest recently because of the studies of the N-terminal region in non-muscle myosins 1b and 1c (which have no SH3 domain), indicating that this region modulates the strain sensitivity of ADP release [70]. Similarly, studies of *Drosophila* muscle myosin have explored the 34 residues following the SH3 domain because it is coded by exon 3, one of four alternately coded exons in the myosin II gene, which in combination, generate all *Drosophila* muscle myosin isoforms and can tune the power output of the muscle [109–111]. Exon 3 codes for a region (residues 69–113) that starts at the last two β-strands of the SH3 domain and includes a 32-amino-acid loop that joins the SH3 to the beginning of the 1st β-strand of the central β-sheet. This region has direct interactions with the purine-binding loop (residues 127–135) on one side and the N-terminal helix (21–30; highlighted in the myosin 1b and 1c study mentioned above). There are also interactions with the ELC on the other side providing what could form a network linking the position of the lever arm and the nucleotide-binding pocket. There are four sequence changes in the exon 3 region following the SH3 domain. These are mostly conservative but they could influence the lever arm-nucleotide pocket communication. There is a double change NA (α) to TP (β) at the end of the purine-binding loop which could also form part of this pathway.

In the long sequence between residues 136 and 294, there is only a single-sequence change (N283D) and those in the short Loop 1 discussed above. Following residue 294, the region encompassing 294–325 is another region that is alternately spliced in *Drosophila* muscle myosin II [110]. This region, coded by exon 7, has 4 sequence changes and has also been shown to influence ADP release from actin–myosin [112]. The region (326–360) following the exon-7-coded region has 6 sequence changes including a group of three residues 348–350, NSM in β changing to AGV in α-myosin. This region is also referred to as the J–K loop (between Helices J and K) and was discussed in detail by Chinthalapudi et al. in relation to non-muscle myosin 2C [113]. The J–K loop provides a connection between Switch 1 in the nucleotide-binding pocket and long Helix-O, which spans the upper 50 kDa domain (see below) with the distal end of the motor domain. Introducing flexibility into the J–K loop of myosin 2c increased the V_max_ of the ATPase threefold, reduced motility fourfold and turned myosin 2c from a low to high duty ratio motor.

There are 5 changes in or near the long Helix-O in the upper 50 kDa domain (417–460). This long helix, which stretches from the actin-binding site (cardiomyopathy loop) to the nucleotide-binding pocket (Switch 1), can link information about the occupancy of the actin- and nucleotide-binding sites. In crystal structures, this helix, along with the major part of the upper 50 kDa domain, appears to move to open and close the major cleft as Switch 1 opens and closes onto the gamma Pi of ATP.

There are no changes following the end of Helix-O (residue 447) until the start of the H–L–H motif (528). There are 6 changes in a region (550–585) that encompasses the part of the H–L–H motif (528–559) and Loop 3 (567–577) both part of the actin-binding site discussed above. Between Loop 3 and Loop 2, there is a region 580–620 with six-sequence changes.

Finally, the whole of the region from after Loop 2 (650) to the end of the converter (790), there are only 5 changes: one just before (S666T), and two just after (T680R and S682A) the third central β-strand, with two more in the converter region (A730V and A744T).

To reiterate, the 76 changes are scattered throughout the motor domain but are concentrated in certain areas. Some of these areas have been subject to detailed study in other myosins, but we are not approaching a complete understanding of the role of each region of the motor in defining motor function. The more myosins we can study at a structural and functional level, the better we will understand the allosteric communication pathways that allow myosin function to be tuned for different biological roles. Not every sequence change between the α- and β-isoform will be tightly coupled to motor function. Some may be required for stability, some to adapt assembly for specific contexts. To understand the significance, if any, of each of these sequence changes for myosin function will require extensive molecular dynamic simulations of this large motor domain. This is not a trivial task for such a large protein although advances in computing power now make such an approach feasible [114–116].

### Comparison of human α- and β-myosin sequence changes with those in orthologous β-myosin sequences

A study of many different orthologous myosins is an alternative route to explore structure–function relationships in myosin. It is known that the contraction velocity of muscle from small animals is faster than that of large animals expressing the same myosin isoform [79]. A study of the isolated myosins from different mammals confirmed that this relationship between size and velocity in motility assays is a property of the myosin isoforms expressed [79]. Johnson et al. [117] argued that a study of the sequences of β-myosins from a large number of mammals may help identify which regions in β-myosin are tuned to adjust the velocity of contraction. From 67 β-myosin sequences (from mouse and bats to elephants and whales), they identified 56 sites which varied in more than 10% of the species examined. In the majority of cases, only two different amino acid residues were found at each location. From these 56 sites, they identified 15 sites that had a significant association with species size (*p* < 0.01) and therefore velocity. A comparison of the sites associated with velocity within β-myosins, and those that differ between human α and β may be informative.

Johnson et al. [117] replaced 12 of the 15 sites in human β with the equivalent sequence for the rat β-myosin to produce a chimeric myosin. They found the velocity of the chimeric myosin in a motility assay was similar to the faster rat, and the ADP release from actin–myosin was also two-fold faster.

Table 2a lists the 15 sites identified by Johnson et al. At each of the 15 sites, the residue differs between large and small mammals, and in each case (except one, I421), human β has the residue expected for a large slower contracting mammal. In contrast, at these same positions, α-human myosin has the same residue as human β at 5 positions, but the residue associated with small faster mammals at 8 sites. At two sites, the residue in the α-isoforms does not match any of those in the β-isoforms (V349, Y421). Therefore, there appears to be an unexpected similarity between human α-myosin sequence and the sequence of β-myosin favoured by small faster mammals.

**Table 2:**
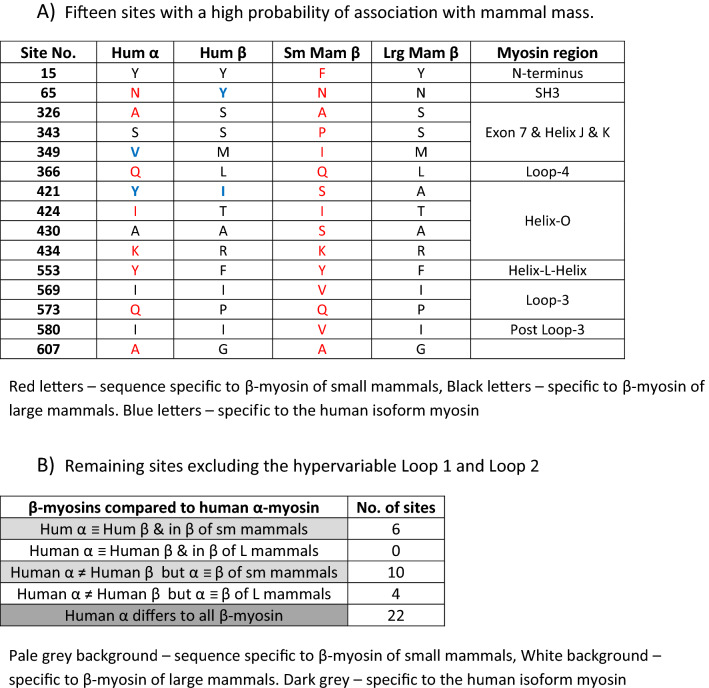
β-myosin sequences that vary between large and small mammals compared to the same site in human α-myosin

A similar analysis of sites that do not have a strong association with mammal size (*p* > 0.05) is shown in Table 2b. Here, the residues in the hypervariable Loops 1 and 2 have been excluded from the analysis as these vary in both length and sequence, leaving 17 sites that differ between large and small mammals’ β-myosin motor domains. Eight of the 17 sites in human α-myosin differ from human β, but are the same as the smaller mammals. Five sites are the same as in both human β and small mammals. Again in human α-myosin, there is a preference for the sequence found in small faster mammals.

It is of interest to note that the regions of β-myosin targeted for sequence changes among species are similar to those that differ between α- and β-myosin, i.e. the N-terminus, the region coded by exons 3 and 7 in *Drosophila*, Helix-O, the actin-binding Loops-3 and -4 and the H–L–H motif. To date, there has been no similar analysis of large and small α-myosin sequences, but there is considerably less variation of sequence with mammal size for α-myosin making any such analysis more difficult. There are also few controlled studies of the maximum velocity of contraction/motility for the α-isoform in different mammals.

## Myofibrils/myocytes expressing α- vs β-myosin

The functional correlates associated with the differential expression of α- and β-myosin isoforms have been indirectly collected over the years from muscle mechanics studies in intact preparations from atrial and ventricular myocardium from humans and animal models that, like humans, express the two different isoforms in the atria and ventricles [80, 118]. Apart from the general difference in the kinetics of the twitch, very little can be associated with the α- and β-myosin function from studies of intact myocardium (see Differences between atrium and ventricle contraction in addition to myosin isoforms). One major difference is in the maximal twitch force, which is lower in the atrium, mostly due to fundamental differences between atrial and ventricular myocardium in myofibril density and in excitation–contraction coupling mechanisms. Also, note the wide spectrum of other sarcomeric protein isoforms besides the myosin motor, discussed in Differences between atrium and ventricle contraction in addition to myosin isoforms.

Attempts made to estimate α and β cross-bridge dynamics from intact multicellular cardiac preparations took advantage of the α/β-isoform shift that had been described following treatment with agents capable of modifying the thyroid state of animal models (usually, rabbit or rat; see above). The mechanical [27, 34, 119, 120], myothermal (rabbit [121, 122]), and ATPase behaviors [27] of papillary muscles of known α- and β-myosin isoform composition were then compared. Note that in these earlier studies, the myosin isoforms were referred to as V1-α, V3-β and V2 intermediate. Results of these studies showed a 3–6 times faster V_max_, 2–3 times faster cycling rate for the α sarcomeres together with about half the economy of contraction compared to β [119, 121, 122]. No difference in the average force per cross-bridge was detected [122].

In more recent years, the functional properties of α- and β-myosins have been characterized in small de-membranated myocardial samples expressing known amounts of the two isoforms. The myosin isoforms expressed were varied in animal model systems (rat, guinea pig, rabbit) via alterations in the thyroid state, while in the case of human and other large mammals, samples from atria and ventricles were compared. In these samples, the mechanics of contraction were studied in calcium-activated tissue strips, myocytes or isolated myofibrils. The various mechanical parameters that can be measured in these studies provide high-resolution tools to estimate the isoform-dependent kinetics and energetics of actomyosin motors working in arrays as cross-bridges throughout the chemo-mechanical cycle (see Fig. 4a). By the simultaneous measurement of force and the ATPase activity of de-membranated cardiac preparations, the energetic cost of isometric tension can be estimated. According to the Huxley 1957 model of contraction, the energetic cost of tension generation is equal to the ATP consumption divided by force, and is directly proportional to the rate at which cross-bridges leave the force generating states [123, 124].

### Human atrial and ventricular strips and myocytes

**Fig. 5:**
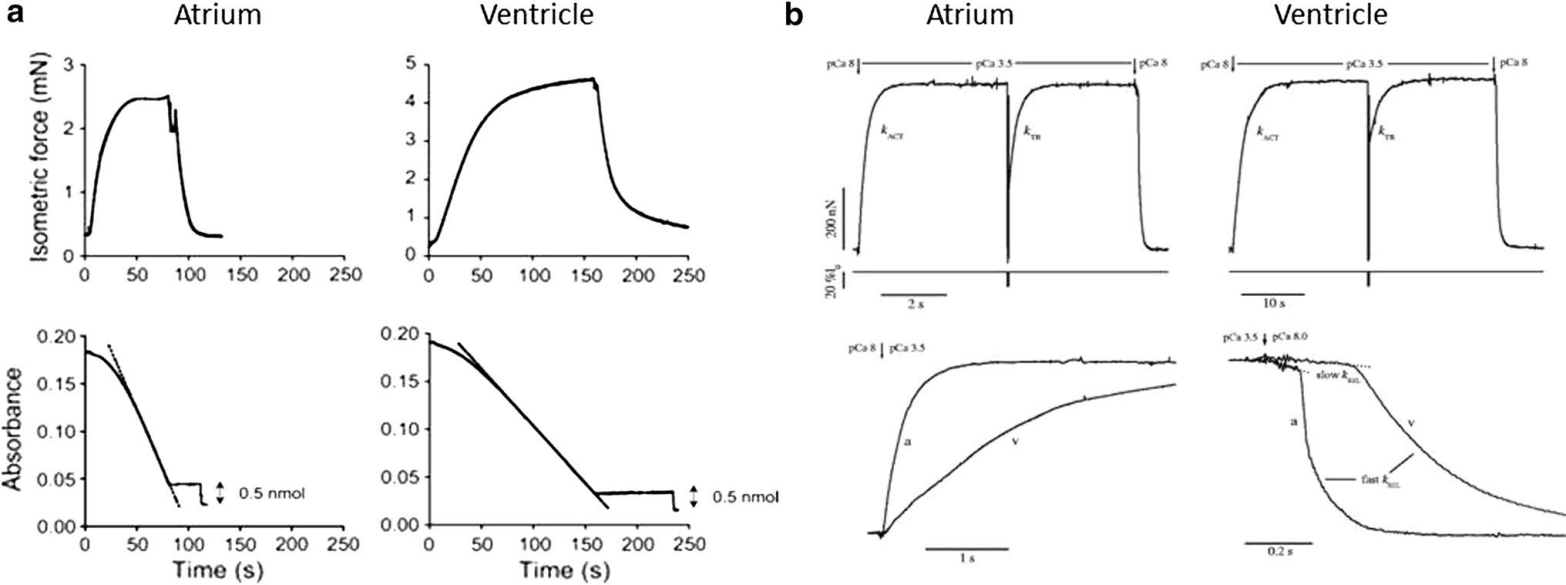
Active tension generation and ATPase activity in human atrial and ventricular de-membranated strips and myofibrils. **a** Recordings of force (upper traces) and absorbance signal of the NADH-coupled ATPase (bottom traces) in a human atrial trabecula (left) and a left ventricular strip (right) during a contraction–relaxation cycle at saturating Ca^2+^ concentration. ATP consumption was determined from the slope of the absorbance signal (15–20 °C); from Narolska et al. REF [125]. **b** Recordings of force responses (upper traces) of atrial (right) and ventricle (left) myofibrils maximally activated and fully relaxed by fast solution switching (pCa changes at *arrows* as indicated (15 °C); from Piroddi et al. [128]. Fast length changes are applied to the myofibrils under conditions of steady tension generation as indicated by the recording of preparation length (lo). *k*_*ACT*_ is the rate constant of tension generation after fast Ca^2+^ activation; *k*_*TR*_ is the rate constant of tension redevelopment after the release–re-stretch. To better resolve the time course of tension activation and relaxation of atrial (a) and ventricular (*v*) myofibrils, the experimental recordings (bottom traces) are superimposed on a faster time base and after normalization for maximal tension. Full tension relaxation is biphasic: slow *k*_*REL*_ is the rate constant of the early slow force decline estimated from the slope of the *regression line* fitted to the tension trace normalized to the entire amplitude of the tension relaxation transient; fast *k*_*REL*_, is the rate constant for the final fast phase of tension decline estimated from a mono-exponential fit

Results from the coupled measurement of maximal calcium-activated isometric force production and ATP consumption in chemically skinned human atrial (75% α) and ventricular (100% β) strips ([125, 126] at 20 °C showed a 3 times larger ATPase in the atria compared to the ventricle (Fig. 5a, Table 3) together with no significant difference in maximal isometric force, P_o_, after correction for myofibrillar density which is lower in the atria. This resulted in a 5 times lower tension cost of contraction (i.e. 5 times more economical contraction) of pure β-myosin human ventricle compared to mostly α human atrial sarcomere (Table 3). The difference in tension cost of contraction of α vs β sarcomeres is preserved also at the physiological, submaximal activation of the myocardium as the calcium sensitivity of force and ATPase activity showed no dependence on MHC isoform (Table 3). (There was also no change in the calcium sensitivity of velocity in a motility assay between purified rabbit psoas vs. porcine cardiac HMM when using recombinant human troponin and tropomyosin [127]). Human α-myosin isoform also imparted 5–10 times higher speed of force development in atrial sarcomeres compared to the almost pure β-isoform of the ventricle, as quantified in skinned myocytes and single myofibrils isolated from human cardiac samples (12–15 °C, [125, 128]). Similar results (force, speed of contraction, ATPase and calcium sensitivity) have been obtained in the many attempts to characterise α vs β sarcomeric functions by studies of skinned ventricular preparations from animal models with altered α/β ratio associated with altered thyroid state [129–131] or different age [132]. Differences in the extent of the overall increase in speed observed in the presence of the α-isoforms were attributed to differences in animal species, temperature and experimental approaches to mechanical measurements. Of note, the difference in sarcomeric functions associated with the presence of α- and β-HC isoforms is further tuned by the expression pattern of atrial and ventricle LC isoforms. This was initially proved to be the case in human right ventricular skinned strips from congenital heart disease patients (100% β-MHC) presenting re-expression of LC1a in adults. This re-expression of LC1a was associated with higher speed of force development and shortening as well as higher maximal force [46] with an overall compensative effect [44, 133]. These conclusions have been confirmed and extended in transgenic mice models showing the increase in maximum shortening velocity and power output of both atrial and ventricular skinned fibers ectopically overexpressing atrial forms of both LC1 LC2 [134].

**Table 3:** Means (± SEM) of mechanical and ATPase parameters of isometrically contracting human atrial and ventricle myofibrils and skinned preparations

	Human Myofibrils^a^		Human skinned muscle^b^	
	Atrial	Ventricular	Atrial	Ventricular
β-MHC content (% of total MHC)	30 ± 5 (8)		24.6 ± 3.2 (14)	94.7 ± 5.2 (6)^$^
Passive tension (mN mm^−2^)	6.4 ± 0.6 (76)	10.1 ± 1.1 (53)^+^		
Active tension				
Po (mN mm^−2^)	125 ± 7 (52)	108 ± 7 (53)	32.8 ± 8.5 (14)	21.1 ± 2.1 (6)
*k*_*ACT*_ (s^−1^)	3.73 ± 0.18 (54)	0.73 ± 0.03 (62)*		
*k*_*TR*_ (s^−1^)	3.55 ± 0.19 (46)	0.68 ± 0.03 (55)*	7.76 ± 1.54 (14)	0.87 ± 0.06 (6)^$^
Full tension relaxation				
Slow phase duration (ms)	126 ± 6 (47)	226 ± 8 (47)*		
Slow k_REL_ (s^−1^)	0.52 ± 0.04 (47)	0.15 ± 0.02 (47)*		
Fast k_REL_ (s^−1^)	16.0 ± 1.0 (47)	2.90 ± 0.16 (47)*		
Ca^2+^ sensitivity of force				
pCa_50_ force pCa_50_ force	5.61 ± 0.03 (8)	5.61 ± 0.02 (8)	5.68 ± 0.04 (14)	5.67 ± 0.04 (6)
nHill force	2.31 ± 0.37 (8)	2.98 ± 0.25 (8)	2.70 ± 0.17 (14)	2.29 ± 0.29 (6)
Ca^2+^ activated ATPase (mmol l^−1^ s^−1^)			0.260 ± 0.025(14)	0.051 ± 0.011 (6)^$^
Ca^2+^ sensitivity of ATPase pCa_50_ ATPase			2.70 ± 0.17 (14)	2.29 ± 0.29 (6)
nHill ATPase			2.33 ± 0.14 (14)	2.36 ± 0.35 (6)
Tension cost (mmol kN^−1^ m^–1^ s^−1^)			11.4 ± 1.4 (14)	2.4 ± 0.3 (6)^$^

Data in parentheses are number of specimens. Data are ^a^from Piroddi et al. [128] and Belus et al. [31] (15 °C) and ^b^ from Narolska et al. [126] (15 °C for mechanical experiments and 20 °C for ATPase assays; Active tension corrected for different myofilament content of atrial and ventricular specimens). pCa_50_ Ca^2+^ concentration at which half the maximal isometric force or ATPase activity is obtained; nHill steepness of the pCa-force and pCa-ATPase relations. Tension cost: ratio of ATPase over isometric tension development. Mechanical parameters as defined in Fig. 5. ^+^ significant at p < 0.002: * significant at *p* < 0.001: $ + significant at *p* < 0.05

### Human atrial and ventricular myofibrils

Studies on isolated myofibrils from human atria and ventricles, activated and relaxed by fast solution switching, provided further understanding of the role of α and β isoforms at the sarcomere level (Fig. 5b; [128, 135, 136]. In these experiments, in addition to the mechanical parameters related to force development (*k*_*TR*_) and redevelopment (*k*_*TR*_), it was possible to clearly resolve the biphasic kinetics of full calcium relaxation [137] and measure the parameters of both the slow and fast relaxation phase (Table 3; [138]). As expected, relaxation kinetics were much slower in human ventricular myofibrils (ca. 100% β) than in the atrial myofibrils (ca. 80% α). For the β-myofibrils, the linear phase of relaxation lasted twice as long and its rate constant, slow k_REL_, was 3–4 times slower than in α-myofibrils [128, 137]. The difference in the rate constant of the fast phase was even larger (Table 3).

A two-fold increase in the half-time of relaxation over controls was also observed in 100% β rat skinned ventricular strips using caged calcium chelators [130]. In myofibril experiments, performed in the virtual absence of inorganic phosphate, slow k_REL_ equals the rate of cross-bridge leaving force generating states [138]. From the five-fold difference in slow k_REL_, observed between human atrial and ventricular myofibrils, a five-fold increase in the rate of cross-bridges leaving their force generating states (associated with MgADP release) can be inferred for α- and β-MHC. A similar conclusion was drawn from direct measurements of the tension cost [125, 131]. Interestingly, sinusoidal length perturbation analysis of LV myocardial strips [139] suggested a four-fold higher rate of MgADP release (and two-fold higher MgATP binding) for 100% α- vs 100% β-HC content of rats and mice generated by an altered thyroid state. Similar results were also obtained in skinned human atrial myocytes [140] and myofibrils [31], from atrial fibrillation patients compared to sinus rhythm controls. Atrial fibrillation caused a significant increase in the relative amount of the slow β-HC expressed by the human atrial myocardium compared to control conditions (from 10–25% to 40–50%, [141]) which could directly account for the observed marked reduction in activation and relaxation kinetics observed in the myofibrils from atrial fibrillation patients [31], in association with the parallel shift of LC1 and LC2 expression from the ventricular to atrial forms [49, 50]. The negative impact of the HC isoform change on the power output and velocity of atrial contraction may then contribute to the atrial contractile dysfunction observed in atrial fibrillation, in the rather complex picture of alterations of gene expression observed in human atrial fibrillation at the proteomic and metabolomics levels [51].

A direct strong positive modulation of power output by the HC isoform ratio was also observed from the force–velocity curves of skinned rat ventricular myocytes [10, 129, 142] showing a linear correlation between the peak of the power profile and the amount of α-isoform over β, up to about 3 times increase for the extreme conditions. Additionally, the key parameters of the force–velocity relation all progressively decreased with increased β-HC content. A decrease in the curvature of the force velocity curve for β-myosin compared to α (for the same load velocity slows proportionately more) is indicative of a change in load dependence of events in the cycle (i.e. ADP release) between the two isoforms. Moreover, the decrease in the values of relative force and velocity at which power output is optimal (F_OPT_ and V_OPT_) with increasing β-myosin content could be a major cause of lower left ventricular power in β-HC heart as striated muscle in vivo is thought to operate at shortening velocities near V_OPT_. The ensemble of these results agrees then with previous measurements in intact preparations as well as with in vitro motility and single molecule studies of α- and β-isoforms. Interestingly, sarcomere length dependence of power output was found to be greater for β- vs α-myosin sarcomeres [10] perhaps indicating a greater impact of the Frank–Starling mechanism in the human ventricle vs atria.

In summary, mechanical studies of cardiac muscle/myocytes/myofibrils expressing known amount of α- and β-myosin showed that the two isoforms working in sarcomeres develop similar maximal isometric force. The same is true for the isometric force developed by atrial and ventricular tissue, after normalization for myofibril content. Mechanical studies, coupled with ATPase measurements in muscle strips, reported marked differences in ATPase and the maximal shortening velocity, V_o_, of α vs β, matching in vitro myosin isoform properties. Modelling of the economy of α vs β (Fig. 4 and Table 1) matches change in LA vs LV tension cost.

Differences in k_ACT_ (dominated by the Force Generation Event) and k_REL_ (dominated by CB detachment—mostly the ADP release step) of α vs β are indicative of isoform-dependent differences in key steps of the CB cycle (Pi release, ADP release). Differences in force–velocity relation, still to be thoroughly assessed, make it possible to argue (Fig. 5 and Table 1) that the load dependent inhibitory effect would be larger on β vs α (V vs A), independent of any difference in motor stiffness.

## Differences between atrium and ventricle contraction in addition to myosin isoforms

**Fig. 6:**
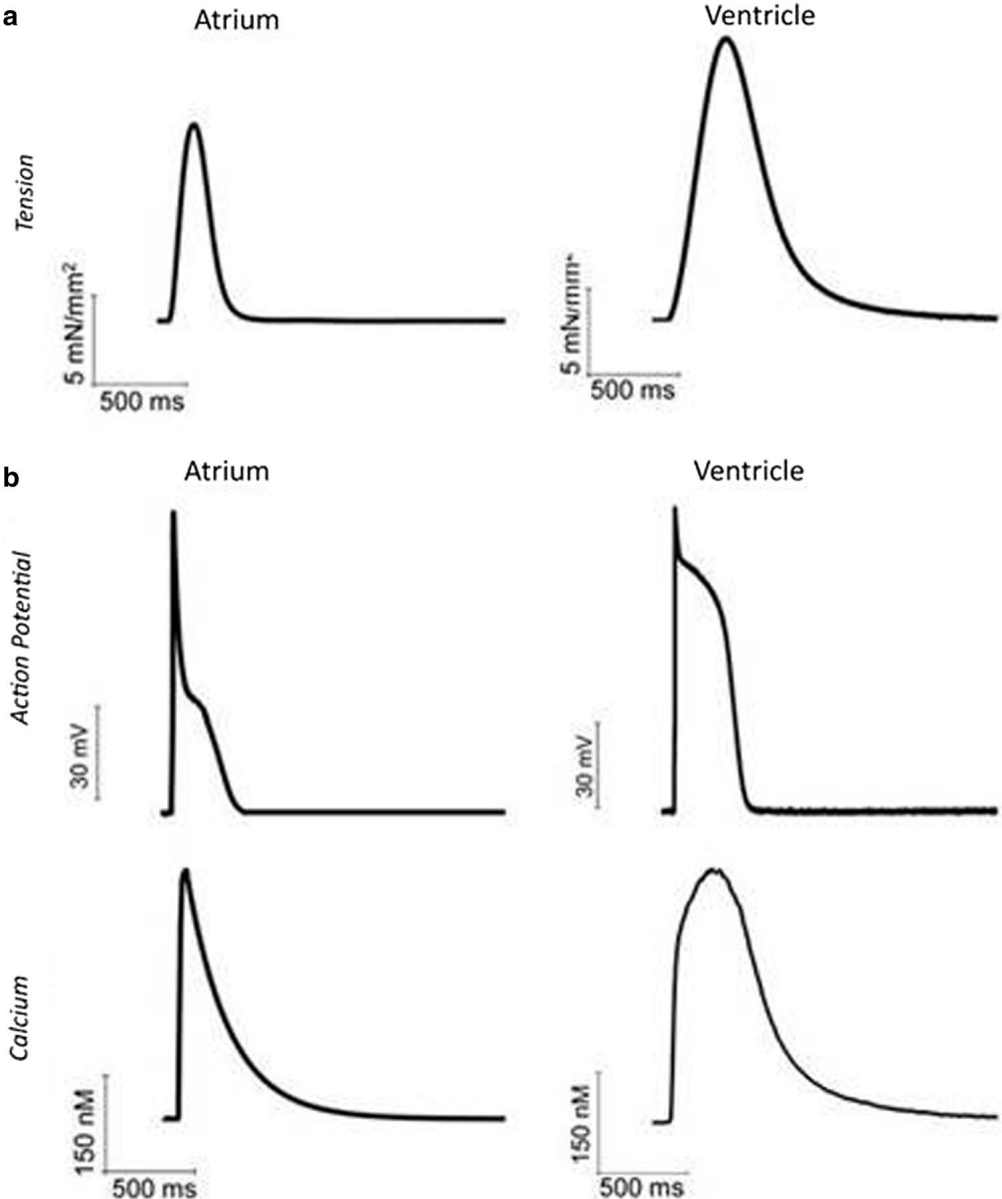
Twitch tension, intracellular calcium transient and action potential of human atrial and ventricular myocardium. **a** Representative isometric twitch tension recordings from intact human atrial and ventricular trabeculae dissected from left atrial and ventricular surgery samples of non-failing non-hypertrophic patients in sinus rhythm. **b** Representative action potentials (top) and intracellular calcium transient (bottom) from human atrial and ventricular cardiomyocytes isolated from the same surgery samples as in a. Ventricular traces are from Coppini et al. [149] Circ 2013 (152). Atrial traces are from Ferrantini and colleagues collected with the same experimental approach as in Coppini et al. [149] Circ 2013 (152)

Contraction twitch amplitude and time course are profoundly different in atrial and ventricular myocardium. Figure 6 shows two examples of twitches recorded from intact human trabeculae, dissected from the left atrium and the left ventricle, respectively. Tension developed by ventricular myocardium is greater (by about 30–50%) and the duration of the entire contraction–relaxation cycle is almost doubled in the ventricle compared to the atrium. These differences are only partly attributable to diversities in myosin isoform as clearly evident from force recordings in the mouse. In this species, ventricular and atrial myocardia express the same myosin isoform, i.e. > 99% α in both chambers, but ventricular contraction is invariably stronger and slower (e.g. see [143]). In atrial myocardium, the extracellular matrix is more abundant and therefore cell density lower. Atrial cardiomyocytes are smaller in volume and typically spindle-shaped (rather than rod shaped). Atrial cells have similar length to ventricular cells yet a transversal dimension that is about half of the ventricular ones (8–10 µm vs 18–20 µm) [144]. In parallel with these histological differences, crucial ultrastructure dissimilarities have been described. First, the atria have a lower density of contractile material in the cytoplasm (with higher relative density of other structures, such as the sarcoplasmic reticulum or mitochondria) [144]. The lower densities of myocytes within the tissue and myofilaments within the cardiomyocytes are likely the main player for the lower tension level developed by atrial myocardium. A second major difference, as mentioned above, consists in the modality and levels of calcium activation, which influence both contraction amplitude and time course [145].

Calcium recordings from human, large mammals and rodent myocardium highlight a faster time course of calcium rise and decay in the atrium of all species (see e.g. [144–149], and Fig. 6b). Responsible for the faster calcium cycling in the atrium are the differences in the expression and function of excitation contraction coupling proteins as well as the different sarcolemmal and sarcoplasmic reticulum (SR) ultrastructural features, including the extent of transverse tubules and abundancy of non-junctional SR (e.g. [150, 151]).

In both atrial and ventricular cardiomyocytes, the bulk of the Ca^2+^ required for contraction is released from the SR in response to a small amount of Ca^2+^entering the cell through the L-type Ca^2+^ channel during the action potential [152]. The action potential duration (APD) and the extent of L-type Ca^2+^ current are known to alter the amplitude and duration of the systolic Ca^2+^transient [153]. The action potential duration is shorter in the atrium (see e.g. [144, 154]. The difference is subtle in small rodents, which have faster heart rates, but severe (up to 200 ms) in the case of human myocardium (Fig. 6b).

In the atrium, the Na^+^–Ca^2+^ exchanger (NCX) protein levels are lower than those seen in the ventricle (e.g. ∼50% less in human atrium vs ventricle [155], and cytosolic Ca^2+^ removal is brought about mainly through Ca^2+^ uptake into the SR by a Ca^2+^-ATPase isoform, SERCA2a. Its activity is modulated by another protein, phospholamban (PLB), which plays an inhibitory role. PLB is less expressed and is coupled to higher SERCA2a levels (e.g. in the atrium of rat and mouse the PLB to SERCA2a ratio is 4–5 times lower than in the ventricle [156]). The lower PLB to SERCA2a ratio underlies the enhanced SERCA2a activity and faster rate of calcium decay of atrial myocardial preparations [143, 157].

The cardiomyocyte membrane is provided with transverse tubules (t-tubules), deep membrane invaginations able to conduct the action potential in depth and therefore responsible for a synchronous activation of calcium release by the SR throughout the entire cell [143, 150, 158]. In the ventricle, particularly in rodents, the transverse tubules are very abundant, they are repeated with a periodicity that is equal to that of the sarcomeres (at the Z lines) and promote a homogeneous and synchronous calcium activation of the whole cell. Conversely, in atrial cells, the density of t-tubules is low or negligible (according to various species) and the SR Ca^2+^ stores can be therefore divided into junctional (j-SR), i.e. the SR regions coupled with surface sarcolemma or t-tubules, and the much more abundant central non-junctional SR (nj-SR). Calcium release from nj-SR occurs through diffusion of calcium from neighboring areas [159]. This non-homogeneous calcium activation in atrial cells (especially at a low inotropic level) implies that myofilaments corresponding to nj-SR are activated at much lower levels and represent a source of contractile material that could be recruited “on demand” [143, 158]. This mechanism could additionally increase the inotropic reserve of the atria, i.e. the potential of increasing Ca^2+^ transient and contraction amplitude in response to need. The real extent of the t-tubule network in atrial myocytes of large mammals (including humans) is still debated, though it is invariably lower compared to that of ventricular myocytes [160–162]. A significant cell-to-cell variability of t-tubule organization and Ca^2+^ homeostasis across the atria has been also reported in large mammals, with the coexistence of homogenously (ventricular-like) activated cells and non-homogeneously activated ones [163]. Concerning the release phase of the Ca^2+^ transient, there also appears to be a greater expression of inositol 1,4,5-tri-phosphate receptors (IP3R’s) in the atrium. In atrial myocytes, sarcolemma IP3R's activation is an additional pathway to trigger SR calcium release that significantly potentiates the RYR2-mediated calcium release and, if activated, results in faster calcium rise and substantial inotropic response. Of note, the rate adaptation of the Ca^2+^ transient and twitch duration in response to inotropic stimuli (i.e. high stimulation frequency and β-adrenergic activation) are less pronounced in the atria because SERCA activity, the main agent responsible for this rate adaptation behaviour, is already extremely high at baseline.

Finally, differences in intracellular Ca^2+^ buffering also play a major role in the amplitude and time course of the activator calcium signal e.g. [164]. In both atrial and ventricular cardiomyocytes, the major ‘fast’ Ca^2+^ buffers are SERCA and the myofilament protein troponin C. Hence, differences in the properties of these intracellular Ca^2+^ buffers and/or their regulation, e.g. different myofilament Ca^2+^ buffering capacity or altered affinity of SERCA for Ca^2+^ through the action of PLB, may also result in alterations of the kinetics of the systolic Ca^2+^ transient between atrial and ventricular myocytes. Though, healthy atrial and ventricular tissue present comparable myofilament calcium sensitivity (Table 3), intracellular Ca^2+^buffering power is substantially greater (up to three times) in the atria compared to the ventricles (e.g. because of higher SERCA activity but also relative mitochondrial density) and this increased Ca^2+^buffering power can contribute to smooth the calcium transient peak.

Besides E-C coupling diversities, a number of differences in the expression of protein isoforms at the sarcomere level can be described in addition to the different myosin isoforms.

Titin transcripts are subjected to a series of differential splicing events in the I-band segment leading to the so-called N2A and N2B isoform transcripts [165]. In most species, small and large titin isoforms are co-expressed in widely varying ratios. The smaller isoform that contains the N2B element (N2B titin) is stiffer than the larger isoform that contains both the N2B and N2A elements (N2BA titin). The expression of small and large titin isoforms at different ratios is thus one means to modulate cardiac myocyte stiffness [166]. The more compliant N2BA titin is more expressed in atria and therefore expected to reduce diastolic tension and facilitate the reservoir function of the atria during their diastole (Fig. 1).

As discussed in Myofibrils/myocytes expressing α- vs β-myosin, the essential (LC1) and regulatory (LC2) Light Chains tune the function of the myosin head and potentially influence its maximum force generating capacity and its Ca^2+^-sensitivity [167–170]. Differences in LC isoforms and their phosphorylation levels as well as chamber-specific post-translational modifications of other sarcomere proteins (e.g. myosin binding protein C, Troponin T) are likely to play a role and considerably complicate the scenario of the mechanisms underlying the differences between atrial and ventricular contraction.

## Outstanding problems

### The super-relaxed state of myosin, and regulation of the thick filament

Thus far, we have avoided mentioning the role of the thick filament in regulating cardiac contractility. This regulatory mechanism has come to prominence in recent years as the discovery of the so-called super-relaxed state of myosin (SRX); the order–disorder transition of the thick filament, and stretch activation mechanisms have coalesced into a novel mechanism for regulating the availability of myosins in the thick filament for contraction (for review see [171–173]. As for other aspects of cardiac contraction, attention to date has focussed on ventricles and β-myosin, so little is known about these mechanisms in atria or with α-myosin. The general assumption is that similar mechanisms will operate but there may be subtle but important differences between the atria and ventricles, and between α- and β-myosin. In a simplified overview, the two heads of a muscle myosin molecule have been observed, in negative stain electron microscopy [174] and cryo-electron microscopy [175, 176], to self-associate such that the actin-binding site of one head blocks the nucleotide pocket of the second head. In this conformation, the ATPase activity is < 10% of the normal, relaxed, switched-off or relaxed myosin in the absence of actin—hence, super-relaxed [177]. These interacting heads are then believed to pack down onto the thick filament to create the ordered thick filament structure observed by both low angle X-ray scattering and electron microscopy [178]. Modulation of the relaxed/SRX myosin and the ordered/disordered thick filaments equilibriums by stretching the thick filament, by Ca^2+^ or by phosphorylation of either the RLC or MyBP-C may regulate contraction output by adjusting the availability of myosin heads. Understanding how α- and β-myosin structures may differ in this respect and how such modulation of myosin availability may differ in atria and ventricles is therefore of interest (see the following section on heterogeneity in myosin dimers).

### Heterogeneity in the myosin content of myocytes: a role for α-myosin in the ventricle of large mammals?

As stated above, the α-myosin accounts for 5–10% of the total myosin expressed in the healthy human LV (e.g. [30]). The functional importance of such low expression levels of α-myosin in the LV is unclear. Genetic studies have associated mutations in the α-myosin gene with some cases of hypertrophic (HCM) and dilated (DCM) cardiomyopathy [179], suggesting that these low levels of α-myosin may be involved in LV contractile function [180]. This may be especially true if α-isoforms are localized to specific regions of the heart. In this regard, it has been shown that the expression of α-myosin in human and pig hearts is more abundant in the sub-epicardium than in the sub-endocardium of the left ventricular free wall [42, 43, 181]. Efficient myocardial pump function depends on the precise coordination and timing of regional electrical and mechanical activation [182], the loss of which significantly impairs heart mechanical function [183, 184]. The transmural distribution of myosin isoforms in the LV of large mammals has been suggested to play an important role in modulating the timing of force generation and relaxation across the ventricular wall by contributing to the twisting untwisting mechanism of LV contraction [43]. Increased expression of α-myosin in the epicardium may accelerate the rate of contraction such that shortening of the earlier-activated endocardial fibres is well coordinated with the later-activated epicardial fibres during systolic ejection. On the other hand, the maintained contraction state of the slow endocardial fibres during iso-volumic relaxation may enhance diastolic filling via stretching of the early relaxed epicardial fibres.

Another factor suggesting a role for the α-myosin in human heart is the observation that there is usually no detectable α-myosin protein in the LVs of failing human hearts e.g. [30]. Conversely, treatment of idiopathic DCM patients with β-blockers resulted in improved cardiac function associated with upregulation of expression of α-myosin [185]. In a related study, rat myocyte fragments expressing small amounts of α-myosin (~ 12%) had a much greater power output (+ 50%) than fragments lacking any α-myosin [129]. These observations suggest that small amounts of α-myosin in a background of β-myosin can have a dramatic effect on function. The presence of α-myosin could therefore result in both faster contraction and greater power of contraction.

This then leads back to questions about the role of small amounts of α-myosin in epicardial fibres in a working heart. In the case where mixed isoforms are present, then questions arise about the local distribution of isoforms in individual thick filament, in sarcomeres and between myocytes. Such questions are hard to address experimentally, but this is an area where modelling studies may provide a route to better understanding of such heterogeneity.

### Heterogeneity in myosin dimers

In the review, thus far, we have considered the myosin as homodimers of α- or β-myosin HC. Yet, in smaller mammals, there is considerable evidence that the αβ heterodimer exists. The three distinct myosin dimers were first discovered by running isolated cardiac myosin in non-denaturing electrophoresis using pyrophosphate gels. The isoforms were referred to as V1 (αα), V2 (αβ) and V3 (ββ) [21] and later identified as the homo- and heterodimers via monoclonal antibodies [186]. There was a period of active research on the three dimers following their identification but little has been published in the last 10 years. The presence of the heterodimer is well established in rats, and guinea pigs where they can be as much as 25% of the total cardiac myosin [27], but there is no evidence of the heterodimers in the human heart or in other larger mammals. However, in the healthy human adult, there is only ~ 5% of α-myosin in the left ventricle, so any heterodimer could only be present at very low levels. This may explain the lack of recent interest in the heterodimers but given that the 5% of α-myosin is confined to small regions the local concentration of α may be much higher and the heterodimer could exist.

Since the myosin tails are 95% identical and the αα- and ββ-myosins must co-assemble in the sarcomere thick filaments, it may not be a surprise that an αβ heterodimer can form [1]. However, there are no reports that the heterodimers can form spontaneously from mature αα and ββ homodimers. Thus, when they form, the heterodimers are likely to assemble at the point of synthesis on the ribosome and, as for homodimers, probably involves specific chaperones during assembly.

The potential of myosin heterodimer formation remains of interest not just for αβ dimers but for myosin mutations responsible for inherited myopathies, i.e. αα or ββ dimers where one chain carries a mutation. Most carriers of such mutations are heterozygous, so both wild-type myosin and myosin carrying the mutation will exist in a myocyte to variable degree depending upon the expression levels of the two isoforms. Thus, the presence of myosin heterodimers is not only possible, but likely given that a mutation in the motor domain may not affect the stability of the dimer. No studies of cardiac myosin heterodimers have been reported for reasons outlined in the next section.

Experimentally, myosin heterodimers have been assembled using smooth-muscle myosin [187] where the two isoforms were expressed in the insect cell expression system each carrying a distinct protein tag. This allowed the heterodimer to be separated from the two homodimers each of which only carried a single type of isolation tag. In this work, the primary interest was in the mechanism of regulation of the 10s/6s switch in smooth-muscle myosin and the interaction between the two heads. A similar approach has been used to study tropomyosin heterodimers, both dimers of different isoforms and dimers carrying cardiomyopathy mutations [188, 189]. Here, the focus was on the effect of the interactions on the stability of the coiled coil. Similar questions arise about the coiled coil of the myosin tail and the packing of the tails into the thick filament where mutations occur in the tail.

The cardiac myosin heterodimers are of interest in the study of cardiomyopathies where most carriers of myosin mutations are heterozygous. Is there a distinct behaviour of myosin carrying one vs. two mutations? Heterodimers are also a potentially useful experimental tool for several reasons. How do the two heads influence each other in the contraction cycle? Can a myosin with only one active head be assembled in a sarcomere? In the switched-off form of myosin, the two heads pack together to form the super-relaxed state (SRX) with very low ATP turnover. A heterodimer would be useful to probe this head–head packing. Furthermore, many mutations associated with HCM are now thought to affect the stability of the SRX. Does this effect occur in a heterodimer or only in the homodimer?

### Is there a difference between human type 1, slow skeletal muscle fibres and ventricle muscle fibres or myofibrils?

As outlined in Differences between atrium and ventricle contraction in addition to myosin isoforms, there are differences between ventricle and atrial myocytes and myofibrils beyond the myosin isoforms. The same is true of cardiac and slow skeletal muscle fibres which both express the same β-myosin isoform but differ in the expression of most other protein isoforms. In humans, biopsies from slow skeletal muscles are far simpler to obtain than from cardiac tissue. This had led to some investigators using soleus muscle biopsies to investigate the behaviour of muscle carrying β-myosin mutations associated with heart diseases [190–194]. With some surprise, however, early mechanical results obtained from soleus biopsies of patients carrying the first discovered HCM-associated mutation in β-myosin (the R403Q mutation [193],) differed considerably from results obtained from ventricular samples of patients carrying the same mutation [195, 196]. Similarly, results from soleus biopsies of patients carrying mutations in the converter region of β-HC [191, 194] differed from those observed in skinned cardiomyocytes from the same patients [197], though, in this latter case, some specific cardiac remodelling associated with the disease had been identified as potential reasons for the reported differences. Differences between the properties of slow skeletal and ventricular β-myosin may, however, exist due to tissue specific post-translational modifications in the β-HC as reported several years ago in one investigation that, unfortunately, remained unfinished because of the untimely death of the first author [198]. Such differences could also explain possible variations in the impact of small molecules targeting the same myosin isoform in different tissues.

## Conclusion

We set out to address the issue of how the two myosin isoforms found in human cardiac tissue are suited their distinct roles in cardiac ventricle vs. atria contraction. As presented, the data on isolated motor domains of myosin isoforms are consistent with the atrial α-isoform (HC + ELC) having a faster ATPase cycle and a faster velocity in a motility assay with a similar duty ratio. This is a result of a faster ADP release rate constant (controlling the lifetime of the attached state and velocity) balanced by a faster ATP hydrolysis step (controlling the lifetime of the detached state) maintaining the duty ratio. The data predict a faster contraction speed as observed in both myocytes and cardiac myofibrils expressing predominantly a single isoform. The higher velocity of the α-isoform means that it is predicted to generate more power (power = force x velocity) than β but at the expense of greater use of ATP. The data on isolated motor domains also predict a change in the ATP economy, α burning more ATP per nm of travel, at maximum velocity and burning more ATP/sec when holding a high force. These myosin isoform properties are consistent with data from human myofibrils and skinned myocytes, as listed in Table 3, where the contribution of EC coupling is removed. Here, k_ACT_, k_TR_, Ca^2+^ activated ATPase and both slow and fast phases of relaxation are 3–five-fold faster for atrial sarcomeres, containing a majority of the α isoform, than ventricular sarcomeres with mostly β-myosin. These data are compatible with each mechanical parameter listed being influenced by the myosin isoform. In the intact electrically stimulated muscle, the relative roles of EC coupling and myosin isoforms are harder to disentangle, both contribute to the differences seen in a typical twitch contraction. This is most clearly seen in the mouse heart where atrial and ventricular twitches are quite distinct despite both containing near 100% α-myosin.

So, what is the role of the myosin isoform if EC coupling has a major role in defining the twitch characteristics? The large variability of β-myosin sequences between mammals of different sizes argues that the β isoform is tightly constrained and matches the needs of the heart (and slow muscle) in a specific organism. The supportive role of α-myosin in the human atrium where its role is to assist refilling the ventricle, (requiring a higher speed of contraction but much less force and, therefore, a lower energy demand than the ventricle) is less constrained.

It is possible that the power that can be generated and the economy of ATP usage are the drivers of isoform diversity. Sarcomeric myosins are normally considered to contract most economically close to the maximum power output (in rat myocytes at 12 °C this is about 30% of P_o_ for α-myosin, 20% P_o_ for β, there are no data for human myocytes or any mammal at 37 °C). But since the human atria actively contract only during its booster function and for a short period (50–100 ms at resting heart rate) against a relatively small blood pressure (well below 20 mmHg), it is likely that the human atrial cells do not contract near maximum power. In contrast, the human β-myosin actively contracts for ~ 3–400 ms in a resting heart, initially an iso-volumic contraction from 10 to 100 mmHg, for ~ 100 ms followed by 200–250 ms at a load of about 100 mmHg pressure as aortic valve opens and the blood is expelled from the ventricle. It seems likely that the economy of ATP usage by the ventricle will be important and that the ventricle will operate near maximum power output. Power and efficiency data on human myocytes are difficult to obtain experimentally although there are data at 30 °C for Type 1 slow skeletal muscle fibres expressing β-myosin [199].

There remain many unresolved questions, as outlined in Outstanding problems, mostly to do with the lack of detailed information on the human atria/α-myosin vs. the more widely studied ventricle/β-myosin. Most of the outstanding questions are amenable to experimental study and we hope that this review may lead to the completion of the experimental work required. Central among the missing information are structures of the α-myosin and actin.α-myosin complexes, together with how a mixed population of α- and β-myosins combine mechanically in the human atrium and parts of the ventricle. Finally, we know that the regulation of the thick filament is different between fast skeletal and cardiac β-myosin/ventricle muscle [200, 201] but little is known about the thick filament regulation in atria/α-myosin.

## Data Availability

Not applicable.
